# Supplementary material for: Initial-site characterization of hydrogen migration following strong-field double-ionization of ethanol
Source: Nat Commun. 2024 Jan 2;15:74. doi: 10.1038/s41467-023-44311-x (PMC10761976; doi:10.1038/s41467-023-44311-x)
Supplement: Supplementary file 1 — Supplementary Information [file 41467_2023_44311_MOESM1_ESM.pdf]

Supplementary information for
Initial-site characterization of hydrogen migration following strong-field double-ionization of ethanol

Travis Severt[1], Eleanor Weckwerth[2], Balram Kaderiya[1], Peyman Feizollah[1], Bethany Jochim[1], Kurtis Borne[1], Farzaneh Ziaee[1], Kanaka Raju P.[1], Kevin D. Carnes[1], Marcos Dantus[3], Daniel Rolles[1], Artem Rudenko[1], Eric Wells[2,*], and Itzik Ben-Itzhak[1]

[1] *J. R. Macdonald Laboratory, Physics Department, Kansas State University, Manhattan, Kansas 66506, USA*
[2] *Department of Physics, Augustana University, Sioux Falls, South Dakota 57108, USA*
[3] *Department of Chemistry, Michigan State University, East Lansing, MI 48824, USA*
(Dated: November 25, 2023)

CONTENTS

1. Supplementary Note 1: Ethanol isotopologues and notation 2
2. Supplementary Note 2: Fragment m/q overlaps and clean fragmentation channels 3
3. Supplementary Note 3: System of equations for complete H_3^+ + $C_2H_3OH^+$ fragmentation channels 4
4. Supplementary Note 4: System of equations for complete fragmentation involving CH_4^+, H_2O^+, and H_3O^+ formation 5
5. Supplementary Note 5: System of equations for incomplete fragmentation involving H_3^+ formation 7
6. Supplementary Note 6: Coincidence Time-of-Flight Data 10
7. Supplementary Note 7: Least-squares fitting procedure 13
8. Supplementary Note 8: Determination of experimental uncertainties 14
9. Supplementary Note 9: Violin plots of the relative site-specific probability for incomplete fragmentation leading to $H_3^+ + C_2H_2O^+ + H$ and $H_3^+ + C_2HO^+ + 2H$ 16
10. Supplementary Note 10: Explanation of the data tabulated in the Source Data files 18
11. Supplementary Note 11: Extension of methodology to molecules with additional hydrogen sites 20
12. Supplementary Note 12: Negligible contributions from ethanol dimers 21
13. Supplementary Note 13: Characterization of isotopic effects 22
    A. Re-normalization 23
    B. Additional Isotopologues 24
    C. The effect of the least-squares fitting 25
    D. Summary 25

Supplementary References 27

1. SUPPLEMENTARY NOTE 1: ETHANOL ISOTOPOLOGUES AND NOTATION

The information provided in this section is similar to what is presented on Figure 1 of the main article and it is repeated here to clearly define the notation used throughout this Supplementary information (SI) document containing the Supplementary Notes (SN). Specifically, the three distinct hydrogen sites in ethanol are labeled in the same manner as in the article and are shown in Supplementary Figure 1 below (which is similar to the cartoons shown in Figure 1 in the main article).

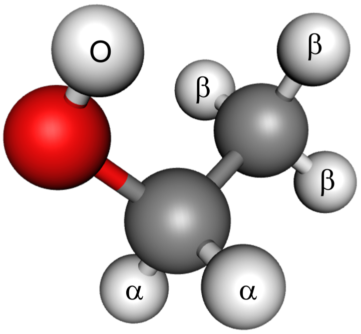

Supplementary Figure 1: Ball-and-stick cartoon of the ethanol molecule with the hydrogen atoms labeled according to their site (α, β, or O) in the manner used in the article and throughout the supplemental information.

To identify each isotopologue without redundancy, we arbitrarily assign each isotopologue a number. The assigned numbers are shown in Supplementary Table I below and are used throughout the article and supplemental information. The number assignments are the same as shown in Figure 1 of the main article.

Supplementary Table I: Isotopologue Details A summary of the number assigned to each isotopologue, the sample source and purity, and the CAS number.

Number	Formula	Purity	Manufacturer	CAS #
1	CH_3CH_2OH	>99.5%	Sigma-Aldrich	64-17-5
2	CH_3CH_2OD	99%	Sigma-Aldrich	925-93-9
3	CD_3CH_2OH	99%	Sigma-Aldrich	1759-87-1
4	CH_3CD_2OH	98%	Sigma-Aldrich	1859-09-2
5	CH_3CD_2OD	98%	CDN Isotopes	22544-49-2
6	CD_3CD_2OH	99.5%	Sigma-Aldrich	1859-08-1
7	CD_3CD_2OD	99.5%	Sigma-Aldrich	1516-08-1

The eighth possible isotopologue, CD_3CH_2OD, was not available at the time of the experiment, and therefore was not used.

All of the ethanol samples were obtained commercially. The vendors and CAS numbers are listed in Supplementary Table I.

2. SUPPLEMENTARY NOTE 2: FRAGMENT m/q OVERLAPS AND CLEAN FRAGMENTATION CHANNELS

The "clean" channels are defined to be fragmentation channels with a mass-to-charge ratio that can be uniquely assigned to a specific ion species. In order to systematically identify the clean channels, we listed the possible fragments at specific m/q values for all measured isotopologues. In Supplementary Table II below, the smaller fragment of each ion-pair is listed. The second detected fragment is not listed in the table, but for complete two-body fragmentation, the second fragment contains all the remaining atoms of the ethanol molecule. For incomplete three-body fragmentation, the missing fragments may contain either one or two hydrogen atoms, meaning each clean fragment listed in Supplementary Table II may be described by multiple equations in the system listed in Eq. S5 of the main article.

Supplementary Table II: Possible fragments at each mass-to-charge ratio. Note that all the ionic fragments are singly charged, therefore for simplicity we listed in the table only the atomic constituents of each fragment ion.

m/q	Iso. 1	Iso. 2	Iso. 3	Iso. 4	Iso. 5	Iso. 6	Iso. 7
	CH_3CH_2OH	CH_3CH_2OD	CD_3CH_2OH	CH_3CD_2OH	CH_3CD_2OD	CD_3CD_2OH	CD_3CD_2OD
1	H	H	H	H	H	H	
2	H_2	H_2<br>D	H_2<br>D	H_2<br>D	H_2<br>D	D	D
3	H_3	H_3<br>HD	H_3<br>HD	H_3<br>HD	H_3<br>HD	HD	
4		H_2D	H_2D<br>D_2	H_2D<br>D_2	H_2D<br>D_2	D_2	D_2
5			D_2H	D_2H	D_2H	D_2H	
6			D_3		D_3	D_3	D_3
12	C	C	C	C	C	C	C
13	CH	CH	CH	CH	CH	CH	
14	CH_2	CD<br>CH_2	CD<br>CH_2	CD<br>CH_2	CD<br>CH_2	CD	CD
15	CH_3	CHD<br>CH_3	CHD<br>CH_3	CHD<br>CH_3	CHD<br>CH_3	CHD	
16	CH_4	CH_2D<br>CH_4	CD_2<br>CH_2D	CD_2<br>CH_2D<br>CH_4	CD_2<br>CH_2D	CD_2	CD_2
17	OH	OH<br>CH_3D	CD_2H<br>OH<br>CH_3D	CD_2H<br>OH<br>CH_3D	CD_2H<br>OH<br>CH_3D	CD_2H<br>OH	
18	H_2O	OD<br>H_2O	CD_3<br>OD<br>H_2O<br>CH_2D_2	OD<br>H_2O<br>CH_2D_2	CD_3<br>OD<br>H_2O<br>CH_2D_2	CD_3<br>OD	CD_3<br>OD
19	H_3O	HDO<br>H_3O	HDO<br>H_3O<br>CD_3H	HDO<br>H_3O	HDO<br>H_3O<br>CD_3H	HDO<br>CD_3H	
20		H_2DO	D_2O<br>H_2DO	D_2O<br>H_2DO	D_2O<br>H_2DO	D_2O<br>CD_4	D_2O<br>CD_4
21			D_2HO	D_2HO	D_2HO	D_2HO	
22			D_3O		D_3O	D_3O	D_3O

3. SUPPLEMENTARY NOTE 3: SYSTEM OF EQUATIONS FOR COMPLETE H_3^+ + $C_2H_3OH^+$ FRAGMENTATION CHANNELS

The following is the overdetermined system of equations used to evaluate complete (two-body) site-specific probabilities for H_3^+ formation. Explicitly, each measured branching ratio is given by the sum of the probabilities associated with the relevant initial-site compositions. These probabilities, in turn, equal the product of the H_3^+ formation probability, $P(H_3^+)$ and the site-specific probability, e.g. $P_{\beta\beta\beta}$. This example is written explicitly as

$$\beta\beta\beta = P(\mathrm{H}_3^+)P_{\beta\beta\beta}\,. \tag{S1}$$

where, as used throughout, β, α, and O are the hydrogen sites in ethanol (see SN 1). Channels that contain overlapping m/q contributions that cannot be isolated are excluded from the set of equations *a priori*, as shown in Supplementary Table II and discussed in SN 6. Notation for the channel branching ratios uses both the mass of the smaller fragment in the channel and the number of the isotopologue. Specifically, we use the $Mi[j]$, where i is the mass to charge ratio of each fragment, j is the isotopologue number as defined in SN 1, and "M" stands for measured branching ratio. For example, $M6[3]$ is the branching ratio of the channel with $m/q = 6$ from Isotopologue 3.

$$\begin{aligned}
M6[3] &= \beta\beta\beta\\
M6[5] &= \alpha\alpha O\\
M5[4] &= \alpha\alpha O + \beta\alpha\alpha\\
M5[5] &= \beta\alpha\alpha + \beta\alpha O\\
M5[6] &= \alpha\alpha O + \beta\alpha O + \beta\beta O\\
M5[3] &= \beta\beta O + \beta\beta\alpha\\
M4[2] &= \alpha\alpha O + \beta\alpha O + \beta\beta O\\
M6[6] &= \beta\beta\beta + \beta\alpha\alpha + \beta\beta\alpha\\
M3[1] &= \beta\beta\beta + \alpha\alpha O + \beta\alpha\alpha + \beta\alpha O + \beta\beta O + \beta\beta\alpha\\
M6[7] &= \beta\beta\beta + \alpha\alpha O + \beta\alpha\alpha + \beta\alpha O + \beta\beta O + \beta\beta\alpha
\end{aligned} \tag{S2}$$

Note that the probabilities in Eq. S2, i.e. $\beta\beta\beta$, $\alpha\alpha O$, etc., are the site-specific probabilities, and are the product of the individual probability of each hydrogen atom in the specific site multiplied by the relevant multiplicity, which is provided explicitly in Supplementary Table III. For example, $P_{\beta\alpha O}$ includes a factor of 6 due to its multiplicity, while $P_{\beta\beta\beta}$ is the same for the site and the individual hydrogen atoms as it has a multiplicity of one.

Supplementary Table III: Multiplicity of H_3^+ ion compositions in equation S2

Variable	Multiplicity
$\beta\beta\beta$	1
$\alpha\alpha O$	1
$\beta\alpha\alpha$	3
$\beta\alpha O$	6
$\beta\beta O$	3
$\beta\beta\alpha$	6

4. SUPPLEMENTARY NOTE 4: SYSTEM OF EQUATIONS FOR COMPLETE FRAGMENTATION INVOLVING CH_4^+, H_2O^+, AND H_3O^+ FORMATION

The following is the system of equations, similar to the one presented in SN 3 for complete fragmentation leading to H_3^+ formation, which are used to determine the site-specific probabilities for H_3O^+, H_2O^+, and CH_4^+ formation in complete fragmentation of ethanol dications. Since the equations for hydronium, water, and methane ions are coupled, we need to solve for all three species simultaneously. Each unknown in the equations is the relative probability of the initial-site composition of one of these species. Three-letters terms (e.g. $\beta\beta\beta$) identify an H_3O^+ composition, while 2-letters (e.g. $\beta\beta$) and 4-letter terms (e.g. $\beta\beta\beta O$) identify the H_2O^+ and CH_4^+ composition, respectively. Notation for the channel branching ratios is the same as used in Eq. S2.

$$
\begin{aligned}
M22[3] &= \beta\beta\beta \\
M22[5] &= \alpha\alpha O \\
M21[4] &= \alpha\alpha O + \beta\alpha\alpha \\
M21[5] &= \beta\alpha\alpha + \beta\alpha O \\
M21[6] &= \alpha\alpha O + \beta\alpha O + \beta\beta O \\
M21[3] &= \beta\beta O + \beta\beta\alpha \\
M20[2] &= \alpha\alpha O + \beta\alpha O + \beta\beta O \\
M22[6] &= \beta\beta\beta + \beta\alpha\alpha + \beta\beta\alpha \\
M19[1] &= \beta\beta\beta + \alpha\alpha O + \beta\alpha\alpha + \beta\alpha O + \beta\beta O + \beta\beta\alpha \\
M22[7] &= \beta\beta\beta + \alpha\alpha O + \beta\alpha\alpha + \beta\alpha O + \beta\beta O + \beta\beta\alpha \\
M19[2] &= \beta\beta\beta + \beta\alpha\alpha + \beta\beta\alpha + \beta O + \alpha O \\
M20[3] &= \beta\alpha\alpha + \beta\alpha O + \beta\beta \\
M20[5] &= \beta\beta\alpha + \beta\beta O + \alpha\alpha + \alpha O \\
M20[4] &= \beta\beta\alpha + \beta\alpha O + \alpha\alpha \\
M19[4] &= \beta\beta\beta + \beta\beta O + \beta\alpha + \alpha O \\
M18[1] &= \beta\beta + \alpha\alpha + \alpha O + \beta O + \beta\alpha \\
M20[6] &= \beta\beta + \beta\alpha + \alpha\alpha + \beta\beta\beta\alpha + \beta\beta\alpha\alpha \\
M19[6] &= \beta O + \alpha O + \beta\beta\beta O + \beta\beta\alpha O + \beta\alpha\alpha O \\
M19[3] &= \alpha\alpha O + \beta\alpha + \beta O + \beta\beta\beta\alpha + \beta\beta\beta O \\
M19[5] &= \beta\beta\beta + \beta\alpha + \beta O + \beta\alpha\alpha O \\
M16[1] &= \beta\beta\beta\alpha + \beta\beta\beta O + \beta\beta\alpha\alpha + \beta\beta\alpha O + \beta\alpha\alpha O \\
M20[7] &= \beta\beta + \alpha\alpha + \alpha O + \beta O + \beta\alpha + \beta\beta\beta\alpha \\
&\quad + \beta\beta\beta O + \beta\beta\alpha\alpha + \beta\beta\alpha O + \beta\alpha\alpha O
\end{aligned} \tag{S3}
$$

Note that the probabilities in Eq. S3 are the site-specific probabilities, and are the product of the individual probability of each hydrogen atom in the specific site multiplied by the relevant multiplicity, which are provided explicitly in Supplementary Table IV.

Supplementary Table IV: Multiplicity of CH_4^+, H_2O^+, and H_3O^+ ion compositions in equation S3

Variable	Multiplicity
$\beta\beta\beta$	1
$\alpha\alpha O$	1
$\beta\alpha\alpha$	3
$\beta\alpha O$	6
$\beta\beta O$	3
$\beta\beta\alpha$	6
$\beta\beta$	3
$\alpha\alpha$	1
$\beta\alpha$	6
βO	3
αO	2
$\beta\beta\beta\alpha$	2
$\beta\beta\beta O$	1
$\beta\beta\alpha\alpha$	3
$\beta\beta\alpha O$	6
$\beta\alpha\alpha O$	3

5. SUPPLEMENTARY NOTE 5: SYSTEM OF EQUATIONS FOR INCOMPLETE FRAGMENTATION INVOLVING H_3^+ FORMATION

Below is the system of equations used to determine the site-specific probabilities for H_3^+ formation in the case of incomplete (three-body) fragmentation. These incomplete fragmentation channels (i.e. three- or four-body breakup), involving H_3^+ formation and elimination of one or two hydrogen atoms, explicitly $H_3^+ + CH_2O^+ + H$ and $H_3^+ + CHO^+ + 2H$ (or H_2), respectively. The set of equations linking the measured branching ratios of different ethanol isotopologues and the site-specific probabilities are listed in Eq. S5 below. The branching ratios were evaluated using normalization to two regions marked by red rectangles on the CTOF plot shown in Supplementary Figure 2. These two regions are defined by the terms in the denominator of Eq. S6. The terms on the right side of each equation in S5 are the site-specific probabilities for H_3^+ formation associated with the elimination of one or two hydrogen atoms. Here too, the probabilities determined by solving Eq. S5 are a product of the H_3^+ formation probability, $P(H_3^+)$, the site-specific probabilities $P_{\alpha\alpha O}$ and $P_{;\beta}$, where the last is the probability for elimination of a hydrogen from the β site, e.g., for M6;1[5] this can be written explicitly as

$$\alpha\alpha O;\beta = P(H_3^+)P_{\alpha\alpha O}P_{;\beta} \ . \tag{S4}$$

The notation used here is similar to the one used for the complete fragmentation channels, where the first three letters of each probability identify the initial-sites of the the H_3^+ composition, while the letters after the semi-colon identify the initial-sites from which the neutral hydrogen is lost. It is important to note that the cases where the site of the lost hydrogen(s) is not determined are denoted by H following the semi-colon (or HH for two-hydrogen loss). The notation for the branching ratios now uses the mass of the smaller fragment in the channel, the mass of the neutral fragment, and the number of the isotopologue. Specifically, we use the $Mi;j[k]$, where i is the mass to charge ratio of the smaller charged fragment, j is the mass of the neutral fragment, and k is the isotopologue number as defined in SN 1. For example, $M6;1[3]$ is the branching ratio of the channel that has a charged fragment with $m/q = 6$ and a neutral hydrogen fragment from Isotopologue number 3.

$$\begin{aligned}
M5;2[6] &= \alpha\alpha O;\mathrm{H} + \beta\alpha O;\mathrm{H} + \beta\beta O;\mathrm{H} \\
M4;1[2] &= \alpha\alpha O;\mathrm{H} + \beta\alpha O;\mathrm{H} + \beta\beta O;\mathrm{H} \\
M6;1[3] &= \beta\beta\beta;\mathrm{H} \\
M6;1[5] &= \alpha\alpha O;\mathrm{H} \\
M5;1[4] &= \alpha\alpha O;\mathrm{H} + \beta\alpha\alpha;\mathrm{H} \\
M3;1[1] &= \beta\beta\beta;\mathrm{H} + \alpha\alpha O;\mathrm{H} + \beta\alpha\alpha;\mathrm{H} + \beta\alpha O;\mathrm{H} + \beta\beta O;\mathrm{H} + \beta\beta\alpha;\mathrm{H} \\
M6;2[7] &= \beta\beta\beta;\mathrm{H} + \alpha\alpha O;\mathrm{H} + \beta\alpha\alpha;\mathrm{H} + \beta\alpha O;\mathrm{H} + \beta\beta O;\mathrm{H} + \beta\beta\alpha;\mathrm{H} \\
M6;1[6] + M6;2[6] &= \beta\beta\beta;\mathrm{H} + \beta\alpha\alpha;\mathrm{H} + \beta\alpha\alpha;\mathrm{H} \\
M5;4[6] &= \alpha\alpha O;\mathrm{HH} + \beta\alpha O;\mathrm{HH} + \beta\beta O;\mathrm{HH} \\
M4;2[2] &= \alpha\alpha O;\mathrm{HH} + \beta\alpha O;\mathrm{HH} + \beta\beta O;\mathrm{HH} \\
M6;2[3] &= \beta\beta\beta;\mathrm{HH} \\
M6;2[5] &= \alpha\alpha O;\mathrm{HH} \\
M5;2[4] &= \alpha\alpha O;\mathrm{HH} + \beta\alpha\alpha;\mathrm{HH} \\
M3;2[1] &= \beta\beta\beta;\mathrm{HH} + \alpha\alpha O;\mathrm{HH} + \beta\alpha\alpha;\mathrm{HH} + \beta\alpha O;\mathrm{HH} + \beta\beta O;\mathrm{HH} + \beta\beta\alpha;\mathrm{HH} \\
M6;4[7] &= \beta\beta\beta;\mathrm{HH} + \alpha\alpha O;\mathrm{HH} + \beta\alpha\alpha;\mathrm{HH} + \beta\alpha O;\mathrm{HH} + \beta\beta O;\mathrm{HH} + \beta\beta\alpha;\mathrm{HH} \\
M6;3[6] + M6;4[6] &= \beta\beta\beta;\mathrm{HH} + \beta\alpha\alpha;\mathrm{HH} + \beta\alpha\alpha;\mathrm{HH} \\
M5;1[3] + M5;2[3] + M5;3[3] &= \beta\beta O;\mathrm{H} + \beta\beta\alpha;\mathrm{H} + \beta\beta O;\mathrm{HH} + \beta\beta\alpha;\mathrm{HH} \\
M5;1[5] + M5;2[5] + M5;3[5] &= \beta\alpha O;\mathrm{H} + \beta\alpha\alpha;\mathrm{H} + \beta\alpha O;\mathrm{HH} + \beta\alpha\alpha;\mathrm{HH} \\
M6;1[6] &= \beta\beta\beta;O + \beta\alpha\alpha;O + \beta\beta\alpha;O \\
M6;2[6] &= \beta\beta\beta;\alpha + \beta\alpha\alpha;\beta + \beta\beta\alpha;\beta + \beta\beta\alpha;\alpha \\
M5;2[6] &= \alpha\alpha O;\beta + \beta\alpha O;\beta + \beta\alpha O;\alpha + \beta\beta O;\beta + \beta\beta O;\alpha \\
M4;1[2] &= \alpha\alpha O;\beta + \beta\alpha O;\beta + \beta\alpha O;\alpha + \beta\beta O;\beta + \beta\beta O;\alpha \\
M6;1[3] &= \beta\beta\beta;\alpha + \beta\beta\beta;O \\
M5;1[3] &= \beta\beta O;\alpha + \beta\beta\alpha;\alpha + \beta\beta\alpha;O \\
M6;1[5] &= \alpha\alpha O;\beta \\
M5;1[5] &= \beta\alpha\alpha;\beta + \beta\alpha O;\beta \\
M5;1[4] &= \alpha\alpha O;\beta + \beta\alpha\alpha;\beta + \beta\alpha\alpha;O
\end{aligned} \tag{S5}$$

(continued from previous page):

$$
\begin{aligned}
M3;1[1] &= \beta\beta\beta;\alpha + \beta\beta\beta;O + \alpha\alpha O;\beta + \beta\alpha O;\beta + \beta\alpha O;\alpha + \beta\alpha\alpha;\beta \\
&\quad + \beta\alpha\alpha;O + \beta\beta O;\beta + \beta\beta O;\alpha + \beta\beta\alpha;\beta + \beta\beta\alpha;\alpha + \beta\beta\alpha;O \\
M6;2[7] &= \beta\beta\beta;\alpha + \beta\beta\beta;O + \alpha\alpha O;\beta + \beta\alpha O;\beta + \beta\alpha O;\alpha + \beta\alpha\alpha;\beta \\
&\quad + \beta\alpha\alpha;O + \beta\beta O;\beta + \beta\beta O;\alpha + \beta\beta\alpha;\beta + \beta\beta\alpha;\alpha + \beta\beta\alpha;O \\
M6;3[6] &= \beta\beta\beta;\alpha O + \beta\alpha\alpha;\beta O + \beta\beta\alpha;\beta O + \beta\beta\alpha;\alpha O \\
M6;4[6] &= \beta\beta\beta;\alpha\alpha + \beta\alpha\alpha;\beta\beta + \beta\beta\alpha;\beta\alpha \\
M5;4[6] &= \alpha\alpha O;\beta\beta + \beta\alpha O;\beta\alpha + \beta\alpha O;\beta\beta + \beta\beta O;\alpha\alpha + \beta\beta O;\beta\alpha \\
M4;2[2] &= \alpha\alpha O;\beta\beta + \beta\alpha O;\beta\alpha + \beta\alpha O;\beta\beta + \beta\beta O;\alpha\alpha + \beta\beta O;\beta\alpha \\
M6;2[3] &= \beta\beta\beta;\alpha O + \beta\beta\beta;\alpha\alpha \\
M5;3[3] &= \beta\beta O;\beta\alpha + \beta\beta\alpha;\beta\alpha + \beta\beta\alpha;\beta O \\
M6;2[5] &= \alpha\alpha O;\beta\beta \\
M5;3[5] &= \beta\alpha\alpha;\beta O + \beta\alpha O;\beta\alpha \\
M5;2[4] &= \alpha\alpha O;\beta\beta + \beta\alpha\alpha;\beta O + \beta\alpha\alpha;\beta\beta \\
M3;2[1] &= \beta\beta\beta;\alpha O + \beta\beta\beta;\alpha\alpha + \alpha\alpha O;\beta\beta + \beta\alpha O;\beta\alpha + \beta\alpha O;\beta\beta + \beta\alpha\alpha;\beta\beta \\
&\quad + \beta\alpha\alpha;\beta O + \beta\beta O;\beta\alpha + \beta\beta O;\alpha\alpha + \beta\beta\alpha;\beta\alpha + \beta\beta\alpha;\beta O + \beta\beta\alpha;\alpha O \\
M6;4[7] &= \beta\beta\beta;\alpha O + \beta\beta\beta;\alpha\alpha + \alpha\alpha O;\beta\beta + \beta\alpha O;\beta\alpha + \beta\alpha O;\beta\beta + \beta\alpha\alpha;\beta\beta \\
&\quad + \beta\alpha\alpha;\beta O + \beta\beta O;\beta\alpha + \beta\beta O;\alpha\alpha + \beta\beta\alpha;\beta\alpha + \beta\beta\alpha;\beta O + \beta\beta\alpha;\alpha O \\
M5;2[3] &= \beta\beta O;\beta + \beta\beta\alpha;\beta + \beta\beta O;\alpha\alpha + \beta\beta\alpha;\alpha O \\
M5;2[5] &= \beta\alpha\alpha;O + \beta\alpha O;\alpha + \beta\alpha\alpha;\beta\beta + \beta\alpha O;\beta\beta \\
\beta\beta\beta;\mathrm{H} &= \beta\beta\beta;\alpha + \beta\beta\beta;O \\
\alpha\alpha O;\mathrm{H} &= \alpha\alpha O;\beta \\
\beta\alpha O;\mathrm{H} &= \beta\alpha O;\beta + \beta\alpha O;\alpha \\
\beta\alpha\alpha;\mathrm{H} &= \beta\alpha\alpha;\beta + \beta\alpha\alpha;O \\
\beta\beta O;\mathrm{H} &= \beta\beta O;\beta + \beta\beta O;\alpha \\
\beta\beta\alpha;\mathrm{H} &= \beta\beta\alpha;\beta + \beta\beta\alpha;\alpha + \beta\beta\alpha;O \\
\beta\beta\beta;\mathrm{HH} &= \beta\beta\beta;\alpha O + \beta\beta\beta;\alpha\alpha \\
\alpha\alpha O;\mathrm{HH} &= \alpha\alpha O;\beta\beta \\
\beta\alpha O;\mathrm{HH} &= \beta\alpha O;\beta\alpha + \beta\alpha O;\beta\beta \\
\beta\alpha\alpha;\mathrm{HH} &= \beta\alpha\alpha;\beta O + \beta\alpha\alpha;\beta\beta \\
\beta\beta O;\mathrm{HH} &= \beta\beta O;\alpha\alpha + \beta\beta O;\beta\alpha \\
\beta\beta\alpha;\mathrm{HH} &= \beta\beta\alpha;\beta\alpha + \beta\beta\alpha;\beta O + \beta\beta\alpha;\alpha O
\end{aligned}
$$

As before, note that the probabilities in Eq. S5 are the site-specific probabilities, and are the product of the individual probability of each hydrogen atom in the specific site multiplied by the relevant multiplicity, which are provided explicitly in Supplementary Table V.

Supplementary Table V: Multiplicity of $H_3^+ + H$ and $H_3^+ + 2H$ compositions listed in Eq. S5. Here also, ";H" and ";HH" denote the cases for which the site the hydrogen atom(s) is eliminated from are undetermined.

Variable	Multiplicity
$\beta\beta\beta;\mathrm{H}$	3
$\alpha\alpha O;\mathrm{H}$	3
$\beta\alpha\alpha;\mathrm{H}$	9
$\beta\alpha O;\mathrm{H}$	18
$\beta\beta O;\mathrm{H}$	9
$\beta\beta\alpha;\mathrm{H}$	18
$\beta\beta\beta;\alpha$	2
$\beta\beta\beta;O$	1
$\alpha\alpha O;\beta$	3
$\beta\alpha\alpha;\beta$	6
$\beta\alpha\alpha;O$	3
$\beta\alpha O;\beta$	12
$\beta\alpha O;\alpha$	6
$\beta\beta O;\beta$	3
$\beta\beta O;\alpha$	6
$\beta\beta\alpha;\beta$	6
$\beta\beta\alpha;\alpha$	6
$\beta\beta\alpha;O$	6
$\beta\beta\beta;\mathrm{HH}$	3
$\alpha\alpha O;\mathrm{HH}$	3
$\beta\alpha\alpha;\mathrm{HH}$	9
$\beta\alpha O;\mathrm{HH}$	18
$\beta\beta O;\mathrm{HH}$	9
$\beta\beta\alpha;\mathrm{HH}$	18
$\beta\beta\beta;\alpha O$	2
$\beta\beta\beta;\alpha\alpha$	1
$\alpha\alpha O;\beta\beta$	3
$\beta\alpha\alpha;\beta O$	6
$\beta\alpha\alpha;\beta\beta$	3
$\beta\alpha O;\beta\alpha$	12
$\beta\alpha O;\beta\beta$	6
$\beta\beta O;\alpha\alpha$	3
$\beta\beta O;\beta\alpha$	6
$\beta\beta\alpha;\beta\alpha$	6
$\beta\beta\alpha;\beta O$	6
$\beta\beta\alpha;\alpha O$	6

6. SUPPLEMENTARY NOTE 6: COINCIDENCE TIME-OF-FLIGHT DATA

Most of the results presented in the main article are derived from the coincidence time-of-flight (CTOF) data, and the basic analysis method has been discussed in earlier publications [1, 2]. This information is commonly examined by plotting the yield as a function of the time-of-flight (TOF) of the first and second ions to arrive at the detector. This is also referred to as a photo-ion photo-ion coincidence (PIPICO) spectra. The laser intensity was chosen to minimize triple ionization, and we excluded the very small number of events with triple coincidences. In Supplementary Figure 2 we show an example CTOF plot for the entire TOF range of the fragment ions of the CD_3CH_2OH isotopologue of ethanol.

The two red rectangles in Supplementary Figure 2 outline the regions of interest for these results. Expanded views of these regions are shown in Supplementary Figure 3, with several channels identified for discussion in the text that follows. The yields of all the dissociation channels with $m_n \leq 6$ are used to evaluate the branching ratios of the "clean" fragmentation channels (See SN 2) used in Eqs. S2-S5, e.g.

$$M6[3] = R_3\left(D_3^+\right) = \frac{N\left(\mathrm{D}_3^+ + \mathrm{C_2H_3O}^+\right)}{\sum\limits_{all} N_C\left(m_1, m_2\right) + \sum\limits_{all; m_n \leq 6} N_{\bar{C}}\left(m_1, m_2; m_n\right)}, \tag{S6}$$

as defined in the main article. In Eq. S6, $R_i(m_1)$ is the branching ratio for the m_1 breakup channel of the ith ethanol isotopologue (as enumerated in SN 1). $N_C(m_1, m_2)$ is the number of measured ion-pairs from the complete channel with m_1 and m_2 being the mass of the first and second ions, respectively. Similarly, $N_{\bar{C}}(m_1, m_2; m_n)$, is the number of measured ion-pairs associated with an incomplete breakup channel with undetected neutral fragment(s) having a mass m_n.

To analyze the yield of the complete two-body fragmentation channels, we calculate the momenta of each fragment in the center-of-mass frame of the breakup (see, for example, Refs. [2, 3]). To limit the contributions of other channels and other possible sources of contamination, we require that momentum conservation is satisfied. To analyze the incomplete three-body fragmentation channels associated with double ionization, we select the apparent ion-pair coincidence channels containing an undetected third atomic fragment. Due to the laser intensity in our present measurements, the triple ionization rate is small, and these rare events are simply removed from the analysis, leaving the incomplete three-body coincidence channel with one or more missing neutral fragment(s). We are interested in the incomplete fragmentation channels with either one or two missing hydrogen atoms, and we note that we cannot distinguish between two separate hydrogen atoms and a single hydrogen molecule, H_2.

While we are primarily interested in the yield of these channels, the momentum of the missing fragment could be calculated if desired. To do this, we compute the initial laboratory-frame velocities of the measured ionic fragments and subtract the average initial center-of-mass velocity of the parent molecule, as determined from the coincidence channels where all fragments are measured. Using momentum conservation, we compute the momentum of the neutral atom. In the case of two hydrogen atoms, this procedure would give the center-of-mass momentum of the pair.

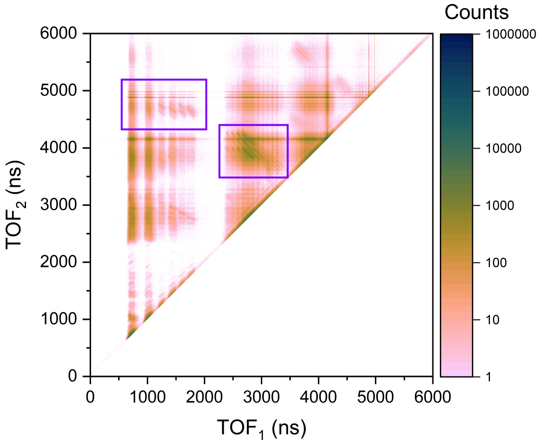

Supplementary Figure 2: The CTOF spectrum (entire TOF range) for CD_3CH_2OH following ionization by laser pulses with 23 fs FWHM duration, a central wavelength of 790 nm, and a peak intensity of 3.0×10^{14} W cm^{-2}. The regions used for normalization of the three-body fragments are outlined by the violet rectangles. An expanded view of these two regions is shown in Supplementary Figure 3. The yield is indicated by the log color scale. Source data are provided as a Source Data file.

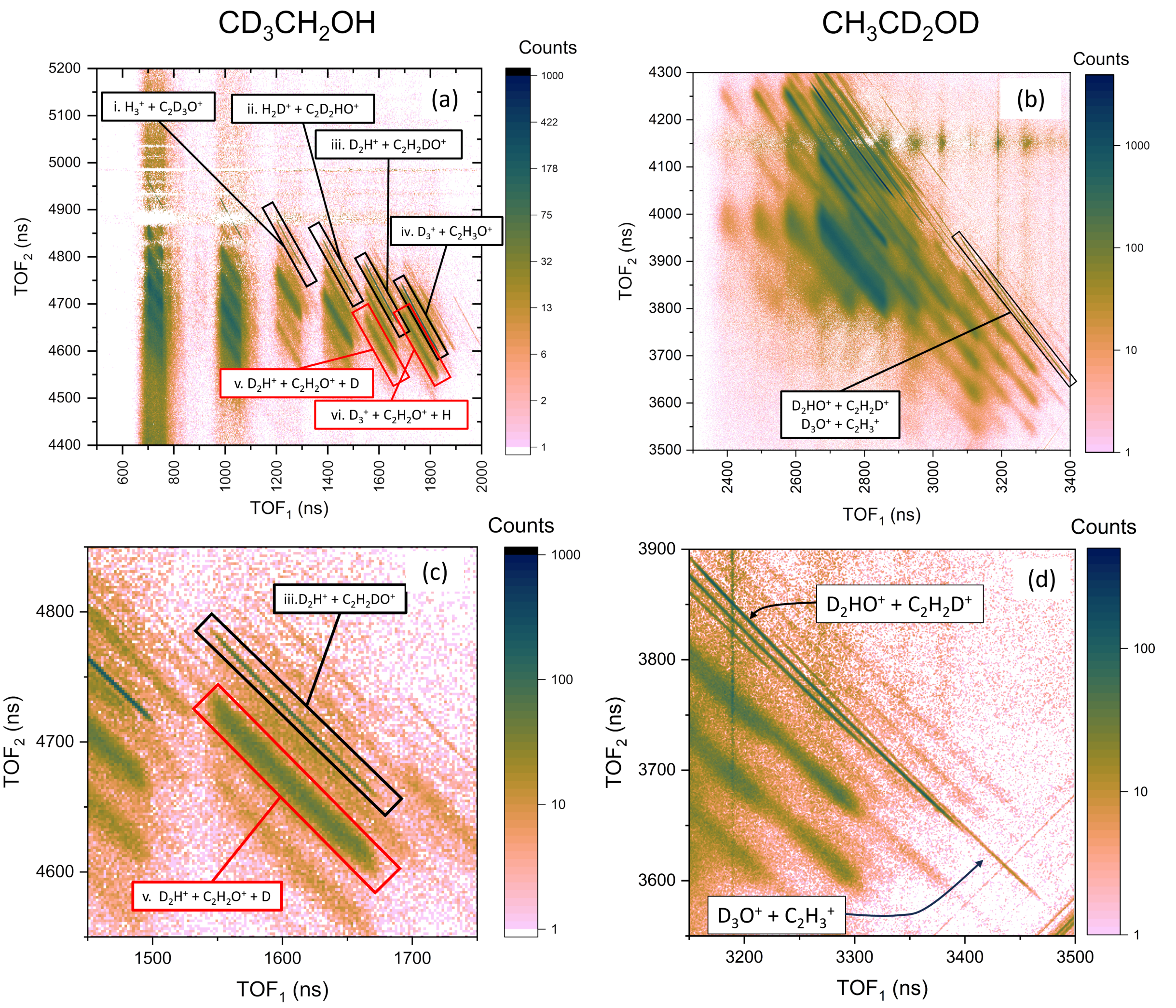

Supplementary Figure 3: Selected regions of the CTOF spectrum for CD_3CH_2OH (left) and CH_3CD_2OD (right) following ionization by laser pulses with 23 fs FWHM duration, a central wavelength of 790 nm, and peak intensity of $3.0{\times}10^{14}$ W cm^{-2}. False coincidences have been subtracted from panel (a) and (b) for better visualization. These regions contain all complete fragmentation channels and all of the incomplete fragmentation channels used in analysis. The yield is shown with a log color scale. (a) CTOF region containing all channels where the smaller fragment has $m/q \leq 6$ atomic mass units and the larger fragment is the remainder of the molecule (complete two-body fragmentation) or else is missing only one or two neutral hydrogen atoms (incomplete fragmentation). Selected channels are discussed in the text and highlighted in black (complete fragmentation) and red (incomplete fragmentation). (b) CTOF region containing fragments with m_1 from m/q = 13 (CH) to m/q = 22, (D_3O). Only two channels are needed from this region for the current analysis: $D_2HO^+ + C_2H_2D^+$ and $D_3O^+ + C_2H_3^+$ which are difficult to separate at the scale shown here. (c) Expanded view of panel (a) showing the ability to separate the complete D_2H^+ + $C_2H_2DO^+$ channel (iii) and the similar incomplete channel (v) that includes an additional hydrogen elimination. (d) Expanded view of panel (b) demonstrating the ability to separate $D_2HO^+ + C_2H_2D^+$ and $D_3O^+ + C_2H_3^+$. Source data are provided as a Source Data file.

To aid visualization in Supplementary Figure 3, false coincidences (also referred to as random coincidences) were subtracted using a global scaling factor matching the main purely random features in the CTOF spectrum (See SN 8). In the actual analysis, however, the false-coincidence subtraction usually employs a scaling factor determined at the level of individual fragmentation channels, and thus is more precise than this figure would indicate. Supplementary Figures 3(c) and 3(d) show additionally magnified regions of interest around some specific coincidence channels. When the channels are complete, they can be further isolated by imposing a momentum conservation requirement.

In Supplementary Figure 3(a), the following channels of CD_3CH_2OH are highlighted:

(i) $H_3^+ + C_2D_3O^+$ - cannot be distinguished from the $HD^+ + C_2D_2H_2O^+$ channel, and thus not used in the fit.

(ii) $H_2D^+ + C_2D_2HO^+$ - cannot be distinguished from $D_2^+ + C_2H_3DO^+$ channel, and thus not used in the fit.

(iii) $D_2H^+ + C_2H_2DO^+$ - no other channel overlaps at these masses, used in the fit of Eq. S2.

(iv) $D_3^+ + C_2H_3O^+$ - no other channel overlaps at these masses, used in the fit of Eq. S2.

(v) $D_2H^+ + C_2H_2O^+ + D$ - cannot be distinguished from $D_2H^+ + C_2DO^+ + H_2$ channel, but both forms are solved for in the fit, so this channel is used in the fit as a part of the sum of all possible probabilities in Eq. S5.

(vi) $D_3^+ + C_2H_2O^+ + H$ - no other channel overlaps at these masses, used in fit of Eq. S5.

The first two channels in the list are examples of channels that are not clean because their mass overlaps with other breakup channels. The other channels (iii-vi) can be matched to inputs of the Eqs. S2 and S5.

In Supplementary Figure 3(b), the expanded region has only two channels of interest for the processes described by Eq. S3, namely $D_2HO^+ + C_2H_2D^+$ and $D_3O^+ + C_2H_3^+$. These two channels come as close together as any pair of complete channels in the analysis. These two channels cannot be discerned at the scale of Supplementary Figure 3(b), although they can be resolved if the plot is magnified further, as shown in Supplementary Figure 3(d). The separation is further aided in the analysis by rotating into time-sum and time-difference coordinates. If needed, there are additional ways to work with overlapping channels, such as exploiting reflection symmetry along the time-of-flight axis. When overalap occurs, it is accounted for in the determination of the uncertainty.

We also note that the number of counts in the region shown in Supplementary Figure 3(b) shows that C-C bond breaking is common in ethanol. Cleavage of the C-C bond, however, rarely resulted in fragmentation channels that we needed to analyze in detail for this work.

7. SUPPLEMENTARY NOTE 7: LEAST-SQUARES FITTING PROCEDURE

A least-squares fitting procedure was implemented with the '`lsq_linear`' function [4, 5] contained in the `scipy.optimize` python package [6]. This function solves a linear least-squares problem with bounds on the variables. In this case, we constrained the fit to prevent negative values for the site-specific probabilities. The `ethanol_leastsq_mc.py` python source code, which is included as an associated text file, can be run with any python package. In this case, we used Jupyter Notebook. The code contains all the input data. In addition, the code performs the procedure to determine the uncertainty in the fit, as described in SN 8.

Since the set of equations is overdetermined, we tested if the fit is sensitive to the use of all the measured branching ratios that can be clearly associated with the breakup channel of interest, which is identified by the mass-to-charge ratio of the charged fragments. These tests were done by removing one or two equations arbitrarily from the equation set and comparing the solutions to each other. In all cases, the changes in the site-specific probabilities were within their errors. We also observed that the R^2 goodness-of-fit was highest when the maximum number of equations was used. Thus, the reported results are obtained with the full set of equations in the fit. For the complete H_3 system (Eq. S2), $R^2 = 0.97$. For the complete CH_4, H_2O, and H_3O system (Eq. S3), R^2 is also 0.97, For the incomplete H_3 system (Eq. S5), $R^2 = 0.88$.

In addition, we verified that increasing the number of iterations in the fitting does not change the results. Furthermore, we examined how the substitution of a branching ratio chosen from a distribution of each branching ratio about its mean with the distribution width determined by the error estimated for that branching ratio affected the results of the fit, as described in SN 8 in more detail.

It is important to note that in some cases the overdetermined set of equations can be associated with a unique hydrogen-rich fragment, like H_3^+ described in SN 3, while in other cases the equations of a few hydrogen-rich fragments are coupled, and therefore they have to be solved together as one overdetermined equation set, for example CH_4^+, H_2O^+, and H_3O^+ described in SN 4.

8. SUPPLEMENTARY NOTE 8: DETERMINATION OF EXPERIMENTAL UNCERTAINTIES

The determination of the branching ratio (Eq. S6) requires the evaluation of the yields of many dissociation channels. Aside from statistical uncertainties, the primary experimental contributions to the uncertainty in the evaluated branching ratio are due to the false coincidence subtraction [2, 7] and the corrections for position dependent losses [1, 2]. As described more fully in SN 12, contributions from ethanol dimers could be safely neglected. Isotopic differences and their contribution to the uncertainty are discussed in SN 13.

As stated in the Methods section in the main article, the false coincidence events that arise from ionization of two different parent molecules by the same pulse were mimicked by randomly pairing individual ion counts from different laser shots, thus generating a sample of purely false-coincidence events. This distribution of false coincidences is then scaled to match a CTOF feature that can arise only from a false coincidence. In the case of complete two-body fragmentation channels and most incomplete three-body fragmentation channels (including all channels that appear in the numerator of Eq. S6), the "random"-scaling factor was determined on a channel-by-channel basis rather than

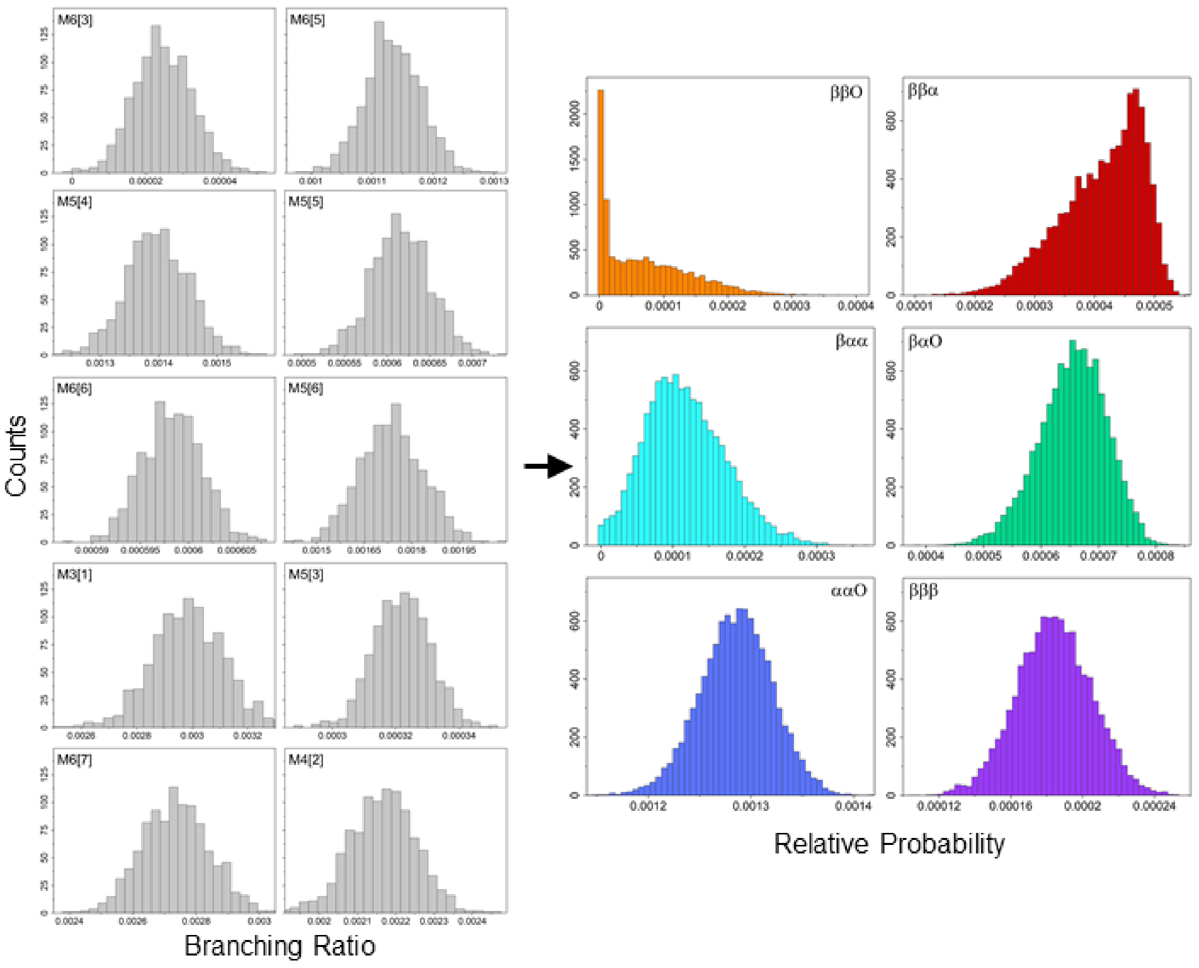

Supplementary Figure 4: (Left) The distributions of the 10 branching ratios used to solve the overdetermined set of equations (i.e., Eq. S2) for the six site-specific probabilities of H_3^+ formation. Each branching ratio distribution contains 1000 values, generated using a Monte Carlo procedure, where the standard deviation of the generated distribution matches the experimental uncertainty of each measured branching ratio. From these distributions, 10^4 sets of the 10 branching ratios are selected randomly, and the least-squares minimization procedure is applied to each set to find the six desired site-specific probabilities. (Right) The output distribution of the 10^4 least-squares fits, where the relative probability is defined by Eq. S6. These output distributions are used to generate the violin plots, shown in Figs. 3 and 4 in the main article, as well as Supplementary Figures 5 and 6 in SN 9. The colors of the right side plots mimic the colors of the initial site composition in Figure 3 and 4 in the main article. Source data are provided as a Source Data file.

using a global random scaling factor, as it is more accurate. Finally, we subtracted the scaled artificially-generated false coincidences from the measured spectrum yielding the true coincidence spectrum [2, 7]. The uncertainty in this procedure, which is mainly due to the error in the random-scaling factor, was evaluated by taking half of the difference between the yields found by using the lowest and highest reasonable random-scaling factors.

Position-dependent losses arise from small regions on the surface of the microchannel plate detector with a nonuniform detection efficiency. We correct the yield for position-dependent losses on our detector using known symmetries about the laser polarization [2]. As with the random-scaling factor, the uncertainty was determined by using half of yield difference obtained by applying the smallest and largest reasonable values of position-dependent loss.

The uncertainties arising from the false-coincidence correction and the position-dependent losses are propagated, along with the statistical (counting) uncertainty, through the branching ratio calculated using Eq. S6. Since most of these procedures involve some degree of human judgement, such as defining the coincidence region of the incomplete three-body fragmentation channels for which momentum conservation cannot be used to unambiguously select an ion-pair event, selecting the best random-scaling factor, or the identification of the boundary of the region with a position-dependent loss, there is potential for systematic error. We evaluated this by selecting one isotopologue and having two different people perform the analysis. Both researchers were experienced in this type of analysis and both used the same analysis code, but they made independent judgements of the parameters described above. By comparing the results of the independent analyses, we concluded that scaling the statistical uncertainty by a factor of seven brought more than 2/3 of the measured branching ratios to within 1σ of each other. While this likely overestimates the actual uncertainty, we believe this conservative approach is warranted due to the coupling of the equations that must be fit to determine the initial-site compositions of the ions.

Error propagation via Monte Carlo methods [8–10], including the evaluation of regression analysis [11], is a standard method of evaluating uncertainty. As outlined in the main article, the uncertainty in the results of the fitting procedure is evaluated by generating, in a Monte Carlo fashion, a normal distribution of 1000 inputs spread around the mean of each measured branching ratio in the system of equations to be solved (Eqs. S2, S3, or S5). The width of the normal input distributions is equal to the experimental uncertainty of each measured branching ratio as determined in the manner described above. For Eq. S2, the ten input distributions are shown on the left side of Supplementary Figure 4. To determine how the uncertainty propagates through the fit of the overdetermined set of equations, a random input is selected from each input distribution. These input values are used in the least squares fit of the relevant equations set (See SN 7). The fitting process generates the site-specific results. The least-squares fit was typically repeated for 10^4 sets of input branching ratios, resulting in the reported values of the site-specific probabilities. For the H_3^+ site-specific system of equations, the output of the 10^4 fitting trials are shown on the right side of Supplementary Figure 4.

Since the relative probability distributions shown in Supplementary Figure 4 do not always result in a Gaussian-like distribution, some care is needed in reporting the experimental uncertainty. For this reason, we describe the results in two different ways. First, the numerical values of the uncertainty are reported using the normal one standard deviation uncertainty (See SN 10). Second, we show violin plots [12] of the probability distributions (See Figs. 3 and 4 in the main article as well as SN 9). A violin plot shows a continuous probability density, thereby giving a visual impression of the full set of fitting results obtained with the Monte Carlo-generated input values. Essentially, the probability density is analogous to a discrete histogram, but is built by summing a series of functions, known as the kernel density function. In the version used in this work, the kernel density function is calculated using the built-in `Ksdensity` function from Origin® plotting software. This function uses a Gaussian kernel as described by Wand and Jones [13]. The violin plots show the median (white circle), the middle 50% of the distribution (black rectangle), and the kernel density function (the curve).

The `ethanol_leastsq_mc.py` python source code, which is included as an associated text file, combines the implementation of the least-squares fit and the Monte Carlo uncertainty analysis. The code can be run with any python package. In this implementation we used Jupyter Notebook. The code contains all the branching ratio input data. Running all three systems with a Monte Carlo size of 10,000 and exporting all the data to delimitated text files takes a Windows-based laptop with a modest CPU around five minutes.

9. SUPPLEMENTARY NOTE 9: VIOLIN PLOTS OF THE RELATIVE SITE-SPECIFIC PROBABILITY FOR INCOMPLETE FRAGMENTATION LEADING TO $H_3^+ + C_2H_2O^+ + H$ AND $H_3^+ + C_2HO^+ + 2H$

Figure 3 of the main article shows the relative site-specific probabilities for complete fragmentation channels of ethanol dications. Since these dissociation channels are complete, there is only a single channel associated with each initial-site composition of H_3^+, CH_4^+, H_2O^+, and H_3O^+. For the incomplete fragmentation channels of H_3^+ shown in Figure 4 of the main article, however, each initial-site composition of H_3^+ may be associated with one or more initial sites of the eliminated hydrogen atom(s) . Examples of these compositions are shown in Figures 4(b) and 4(d) of the main article. In this SN, we report the incomplete fragmentation channels of H_3^+ not shown in the main article.

Of the six H_3^+ initial-site compositions, shown in Figure 4(a) of the main article, five have multiple possibilities for the initial site from which the neutral hydrogen is eliminated. The $H_3^+ + C_2H_2O^+ + H$ fragmentation channel can be associated with a neutral hydrogen from the α, β, or O initial sites, as shown in Figure 4(b) of the main article. Four initial-site compositions of $H_3^+ + H$ each have two possibilities for the initial site of the neutral hydrogen atom. The relative probabilities of three of these cases are shown in Supplementary Figure 5. The remaining $H_\beta H_\alpha H_O + H$ initial-site composition, in which the hydrogen elimination can be from the α or β sites, has a probability consistent

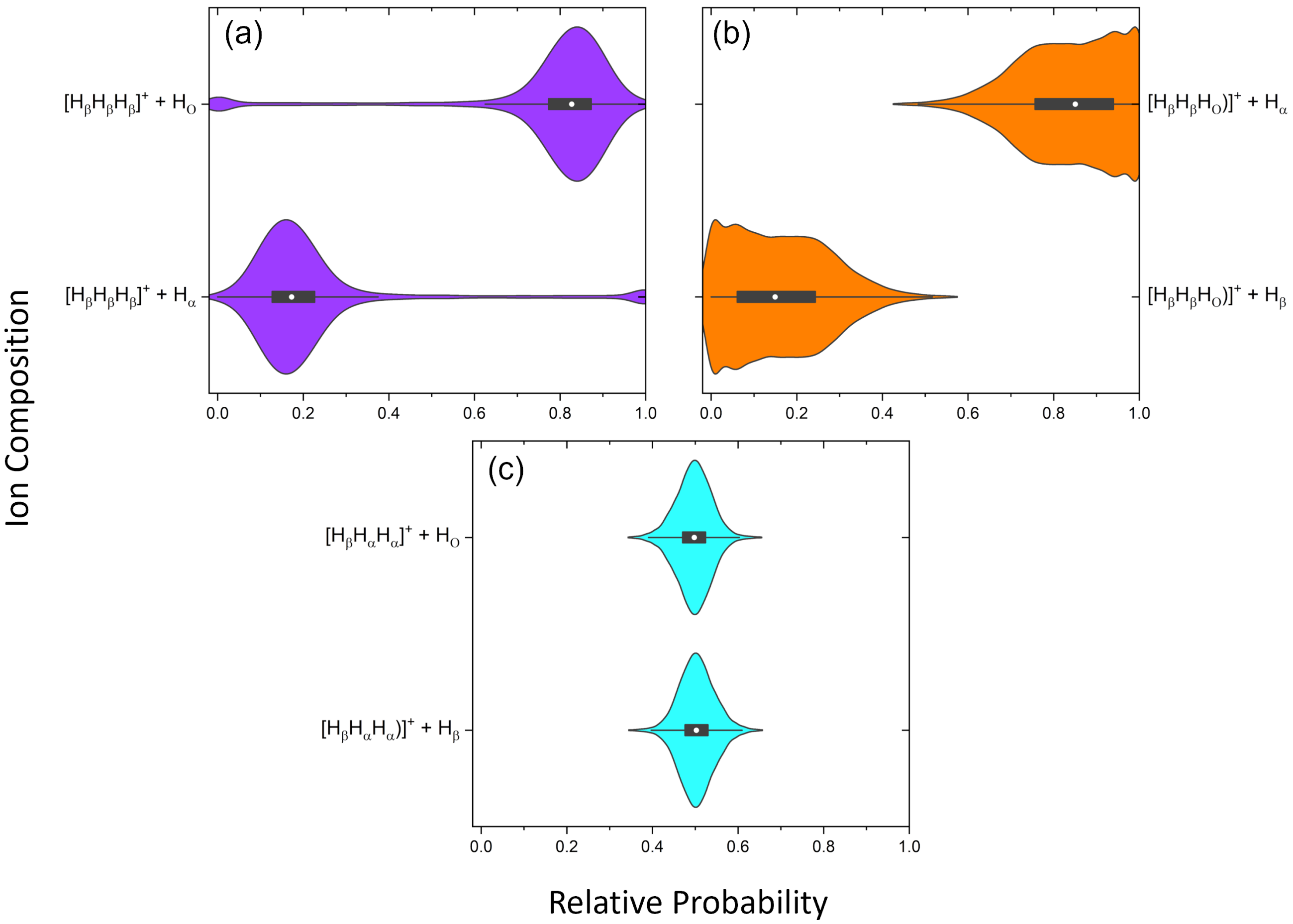

Supplementary Figure 5: Violin plots [12] of the relative site-specific probabilities for incomplete fragmentation channels of ethanol dications leading to $H_3^+ + C_2H_2O^+ + H$ with the initial site of the neutral hydrogen identified. (a) The $H_\beta H_\beta H_\beta + H$ initial-site composition, in which the hydrogen loss can be from the α or O sites. $P(H_\beta H_\beta H_\beta\ ,H) = (1.60{\pm}0.32){\times}10^{-4}$. (b) The $H_\beta H_\alpha H_O + H$ initial-site composition, in which the hydrogen loss can be from the α or β sites. $P(H_\beta H_\beta H_O\ ,H) = (4.51{\pm}0.71){\times}10^{-4}$. (c) The $H_\beta H_\alpha H_\alpha + H$ initial-site composition, in which the hydrogen loss can be from the β or O sites. $P(H_\beta H_\alpha H_\alpha\ ,H) = (7.01{\pm}0.65){\times}10^{-4}$. As has been the practice in the main article, the numerical value of the uncertainties quoted in this caption are 1σ values obtained using normal statistics, although the distributions may be non-Gaussian, as indicated by the violin plots. The shading colors of the plots mimic the colors of the initial site composition in Figure 3 and 4 in the main article. Source data are provided as a Source Data file.

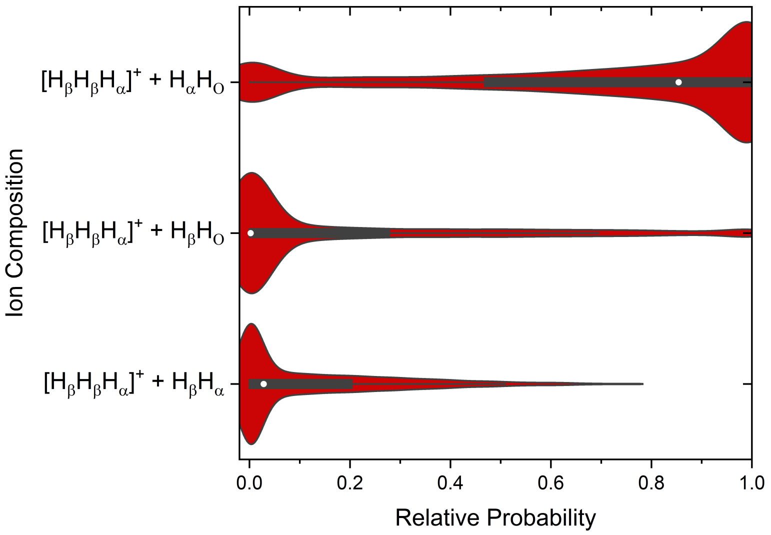

Supplementary Figure 6: Violin plot [12] of the relative site-specific probabilities for incomplete fragmentation of ethanol dications leading to $H_3^+ + C_2HO^+ + 2H$ with the initial sites of the neutral hydrogen atoms identified. Specifically, the above plot shows the $H_\beta H_\beta H_\alpha +$ 2H initial-site composition with $P(H_\beta H_\beta H_\alpha\ ,2H) = (5.5{\pm}2.3){\times}10^{-5}$. The other channels with non-zero probability for $H_3^+ + 2H$ are shown in Figure 4(d) of the main article. As has been the practice in the main article, the numerical value of the uncertainty quoted in this caption is the 1σ value obtained using normal statistics, although the distributions may be non-Gaussian, as indicated by the violin plots. The shading colors of the plots mimic the colors of the initial site composition in Figure 3 and 4 in the main article. Source data are provided as a Source Data file.

with zero, specifically $P(H_\beta H_\alpha H_O\ ,H) = (3.8{\pm}5.3){\times}10^{-5}$. The uncertainty quoted here, as is the case throughout this SN, is the 1σ value obtained using normal statistics. The $H_\alpha H_\alpha H_O + H$ initial-site composition, in contrast, can only be associated with hydrogen elimination from the β site.

When two hydrogen atoms are eliminated in the incomplete fragmentation, there are 12 initial-state compositions that can occur. Similar to the case where a single hydrogen is eliminated, the $H_\alpha H_\alpha H_O + 2H$ initial-state composition can only be associated with the elimination of two hydrogen atoms from the β site. Figure 4(d) of the main article shows the violin plots for the $H_\beta H_\beta H_\alpha^+ + C_2HO^+ + 2H$ and $H_\beta H_\beta H_O^+ + C_2HO^+ + 2H$ initial state compositions. The violin plots of the remaining $H_3^+ + 2H$ fragmentation channel is shown in Supplementary Figure 6. Within a 1σ uncertainty, both the $H_\beta H_\beta H_\beta^+ + 2H$ and $H_\beta H_\alpha H_O^+ + 2H$ initial-site compositions are consistent with zero, with $P(H_\beta H_\beta H_\beta\ ,2H) = (0.8{\pm}1.5){\times}10^{-5}$ and $P(H_\beta H_\alpha H_O\ ,2H) = (1.5{\pm}2.2){\times}10^{-4}$, respectively. The $H_\beta H_\beta H_\alpha^+ + 2H$ initial-site composition, which is shown in Supplementary Figure 6, is most likely to be formed in association with hydrogen elimination from the α and O sites.

10. SUPPLEMENTARY NOTE 10: EXPLANATION OF THE DATA TABULATED IN THE SOURCE DATA FILES

Tabulated values of the relative probabilities of the initial-site composition of all the ions evaluated in this work are given in the associated spreadsheet file `Source Data - Branching Ratios.xlsx`. These branching ratios form the experimental results that are used to fit the systems of equation described in SN 3-5. To avoid potential character translation issues, the standard Greek letter notation, shown in Supplementary Figure 1 and used throughout the article and the SI, has been altered according to the following scheme:

- $\beta = X$
- $\alpha = Y$
- $O = Z$.

In the first sheet, (`site-specific probabilities`) three different relative probabilities are listed. `Probability relative to three-body` (Column F) is defined using Eq. S6 and is the relative probability used in the article. The associated uncertainty of this relative probability is listed in Column G.

In the case of complete (two-body) fragmentation channels, another reasonable way of defining the relative probabilities is by normalizing the branching ratios to only the sum of complete (two-body) fragmentation channels. For example, the analogous branching ratio to Eq. S6 would now be

$$R_3^{two-body}\left(D_3^+\right) = \frac{N\left(\mathrm{D}_3^+ + \mathrm{C_2H_3O}^+\right)}{\sum\limits_{all} N_C\left(m_1, m_2\right)}, \tag{S7}$$

where $R_i^{two-body}(m_1)$ is the branching ratio for the m_1 breakup channel of the ith ethanol isotopologue as enumerated in SN 1 and $N_C(m_1, m_2)$ is the number of measured ion-pairs from complete channels with m_1 and m_2 being the mass of the first and second ions. This probability is called `Probability relative to two-body` and is listed in Column D, and the associated uncertainty is given in Column E.

Finally, the relative probability of a particular initial-site composition can be determined relative to all possible ion compositions for that fragment. By definition, these probabilities all sum to one for each fragment and are shown in the violin plots. The spreadsheet identifies these as `Probability relative to fragment` in Column B and the associated uncertainty is listed in Column C.

The spreadsheet lists eight sets of probabilities. The description of each data set is listed in Supplementary Table VI.

Supplementary Table VI: Probabilities Listed in Associated Spreadsheet

Label	Description
H3	$\mathrm{H_3^+} + \mathrm{C_2H_3O^+}$ complete two-body fragmentation
H3O	$\mathrm{H_3O^+} + \mathrm{C_2H_3^+}$ complete two-body fragmentation
H2O	$\mathrm{H_2O^+} + \mathrm{C_2H_4^+}$ complete two-body fragmentation
CH4	$\mathrm{CH_4^+} + \mathrm{CH_2O^+}$ complete two-body fragmentation
H3/H	$\mathrm{H_3^+} + \mathrm{C_2H_2O^+} + \mathrm{H}$ incomplete three-body fragmentation, H initial site not identified
H3/HH	$\mathrm{H_3^+} + \mathrm{C_2HO^+} + 2\mathrm{H}$ incomplete three-body fragmentation, 2H initial sites not identified
H3/H(ss)	$\mathrm{H_3^+} + \mathrm{C_2H_2O^+} + \mathrm{H}$ incomplete three-body fragmentation, H initial site indentified
H3/HH(ss)	$\mathrm{H_3^+} + \mathrm{C_2HO^+} + 2\mathrm{H}$ incomplete three-body fragmentation, 2H initial sites identified

The next three sheets contain all of the measured branching ratio data. `2 body (complete) wrt 2 body` has the measured branching ratios with respect to all complete two body fragmentation for all seven isotopologues. Each isotopologue takes up two columns, the first column `BR` is the branching ratio itself, while the second column `BR unc` is the uncertainty. The associated error propagation code multiplies these numbers by seven to get the sum of the statistical and systematic errors as discussed in SN 8. The next sheet `2 body (complete) wrt all counts` is the same data but with respect to the sum of the two-body and three-body counts.

The sheet `3 body wrt all counts` has the branching ratios of interest for the three-body data presented in the main article. Unlike the previous two sheets, these uncertainties represent the sum of the statistical and systematic uncertainty. These three sheets represent the input data needed to create Figures 3 and 4 of the main article using the `ethanol_leastsq_mc.py` python code contained in the supplemental information package.

The file `Source Data - Figures.xlsx` contains the source data for figures 2-5 of the main article. Each dataset is listed in a separate sheet in the Excel spreadsheet. Column headers describe the data, which is almost exclusively probabilities of particular dissociation products.

Likewise, the file `Source Data - Suplementary Figures 3-12.xlsx` contains the source data for Supplementary Figures 3 - 10. Each dataset is listed in a separate sheet in the Excel spreadsheet. The time-of-flight data in sheets `SI-Fig3a`, `SI-Fig3b`, `SI-Fig3c`, `SI-Fig3d` have 2 ns bins. Sheets `SI-Fig7` and `SI-Fig8` have 1 ns bins. For the other sheets, which contain probabilities of particular dissociation products, the data is labelled with headers.

The full CTOF data shown in Supplemental Figure 2 produces an Excel file that is too large for the supplemental information guidelines, so it is saved as a tab-delimited ascii file, `Supplementary Figure 2.txt`. This data has a bin size of 2 ns.

11. SUPPLEMENTARY NOTE 11: EXTENSION OF METHODOLOGY TO MOLECULES WITH ADDITIONAL HYDROGEN SITES

A relevant question is whether this technique of measuring the branching ratios for many isotopologues, and then solving the overdetermined set of equations by a least-square fit algorithm to obtain the relative probabilities of each initial-site composition, could be applied to larger molecules as well. As the hydrogen-rich molecule grows, the number of possible initial-site compositions of H_3^+ increases, which expands the number of unknown probabilities to solve for in the system. However, larger molecules with more hydrogen sites also allow more deuterium-tagged isotopologues to be created and tested. If we assume that any isotopologue with either entirely deuterium or entirely hydrogen atoms at each site can be synthesized, the number of available isotopologues for a molecule with n hydrogen sites is 2^n. The equation system can be solved, using the least-square fit approach, if the number of equations is greater than the number of initial-site compositions leading to H_3^+ formation.

As long as the molecule is comprised of only C, H, and O atoms, there are no additional mass overlaps at 6 a.m.u. or 5 a.m.u. for any isotopologue. It turns out that any isotopologue with at least 3 deuterium atoms and at least 1 hydrogen atom will have exactly 2 clean channels and thus 2 equations, and any other isotopologue will have exactly 1 clean channel and thus 1 equation. Therefore, the number of equations always exceeds the number of isotopologues. The number of possible H_3^+ initial-site compositions is slightly more difficult to generalize, but can be worked out in specific cases. If we define t to be the number of 3H sites in the molecule (that is, the number of primary carbon sites), d to be the number of 2H sites in the molecule (secondary carbon sites), and s to be the number of 1H sites in the molecule (tertiary carbons or hydroxyl groups), and h to be the total number of hydrogen or deuterium atoms in the molecule, that is, $h = 3t + 2d + s$, we can find that the number of possible initial-site compositions for a non-symmetric molecule (i.e., a hydrocarbon chain with different edge atoms) is given by

$$\begin{aligned}\#\text{ of site combinations} = &\binom{h}{3} - d\binom{s}{2} - 3s\binom{d}{2} - 2\binom{d}{2} - 7\binom{d}{3} - 5tds - 2t\binom{s}{2} - 2td \\ &- 11t\binom{d}{2} - 2ts - 8s\binom{t}{2} - 5td - 17d\binom{t}{2} - 16\binom{t}{2} - 26\binom{t}{3},\end{aligned} \tag{S8}$$

where $\binom{m}{k}$ is the standard "m choose k" defined by

$$\binom{m}{k} = \frac{m!}{k!(m-k)!}, \tag{S9}$$

with the added stipulation that $\binom{m}{k} = 0$ if $m < k$. A formal proof of this is mathematically straightforward but tedious and is thus not included here.

The result of this determination is that as the molecule grows, the number of equations grows faster than the number of unknown probabilities.

While it is not as straightforward to generalize the result to any molecule composed of carbon, hydrogen, and oxygen without making assumptions about the structure, it is not too difficult to work it out for a few specific cases of interest, such as 2-propanol. A few molecules, which can be counted easily, are shown in Supplementary Table VII as examples.

Supplementary Table VII: Examples of extensions to larger molecules

Molecule	Structure	Isotopologues	Equations	3H Combinations
1-propanol	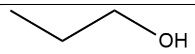	16	27	14
2-propanol	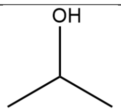	16	27	12
1-butanol	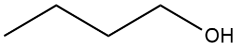	32	58	27
hexan-3-one	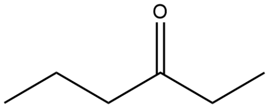	32	59	32
1-pentanol	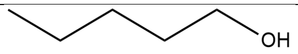	64	121	46
1-hexanol	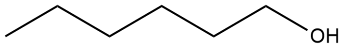	128	248	72

12. SUPPLEMENTARY NOTE 12: NEGLIGIBLE CONTRIBUTIONS FROM ETHANOL DIMERS

It has been known for some time that when gas is expanded into a vacuum, small clusters (dimers, trimers, etc.) can form [14, 15]. In our experiment, the presence of ethanol dimers would present a difficulty for our analysis of the incomplete fragmentation channels, since momentum conservation cannot be used to verify that the fragments detected in coincidence originate from a single ethanol monomer. To avoid this potential problem, the target ethanol gas was not seeded in a carrier gas and thus the driving pressure of the jet of the COLTRIMS setup was quite low, limiting dimer formation at the expense of a slightly warmer target. The absence of significant ethanol dimers was verified from the time-of-flight spectrum, shown in Supplementary Figure 7. From the time-of-flight spectrum, we evaluated the ratio of dimer cations to monomer cations to be about 10^{-4}. For example, we measured $(1.204{\pm}0.086){\times}10^{-4}$ for the CH_3CH_2OH target shown below. In addition, the CTOF spectra showed essentially no true coincidences between two ethanol monomer cations, as shown in Supplementary Figure 8. As a result of these measurements, we could justify neglecting contributions from dimer, or larger, ethanol clusters in the analysis.

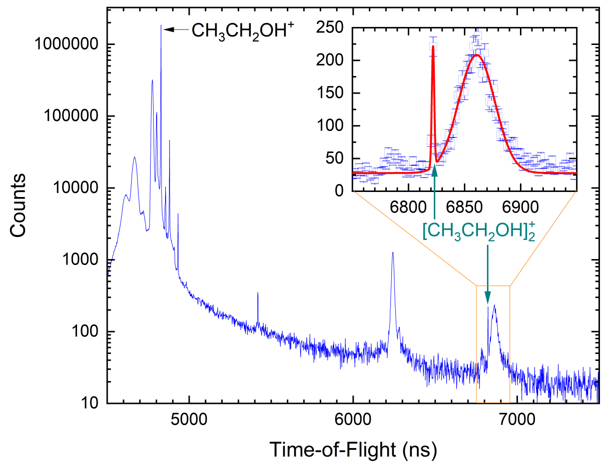

Supplementary Figure 7: High-mass region of the time-of-flight spectra for the CH_3CH_2OH (isotopologue #1) target, showing the relative magnitude of the monomer cation and the dimer cation on a log scale. The narrow peak associated with the dimer cation at 6821 ns ($m/q = 92$) is partially overlapping with another background peak. A two-Gaussian fit was used to separate the contributions from the two sources, as shown in the inset. Source data are provided as a Source Data file.

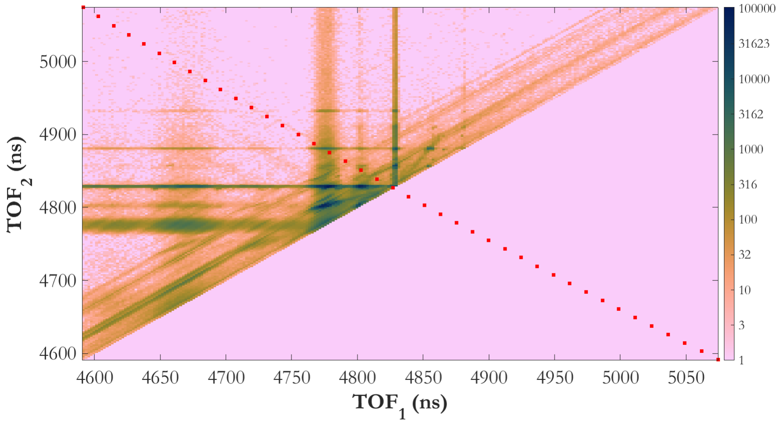

Supplementary Figure 8: The region of the CTOF spectra that would include coincidences between two ethanol monomer cations, as indicated by the dashed red line. The rate of these coincidence events is below our detection sensitivity. Source data are provided as a Source Data file.

13. SUPPLEMENTARY NOTE 13: CHARACTERIZATION OF ISOTOPIC EFFECTS

As described in the Results section and Figure 2 of the main article, the fundamental assumption of deuterium substitution is that the mass difference between ^{1}H and ^{2}D does not significantly affect the properties to be studied. We compared the branching ratios of several relevant channels in the CH_3CH_2OH and CD_3CD_2OD isotopologues to evaluate the validity of this assumption. Further analysis of the deviations in these branching ratios gives a more complete picture of the overall magnitude of isotopic effects in this data set and indicates that neglecting isotopic differences does not change the analysis significantly.

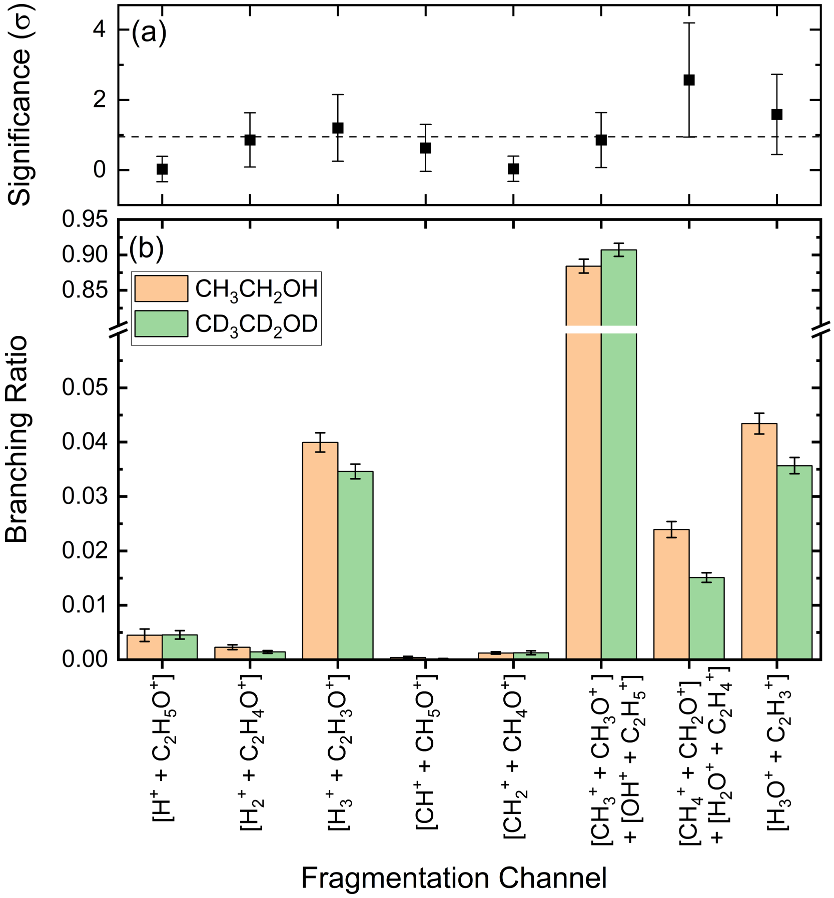

Supplementary Figure 9: (a) The significance (see Eq. S10) of the difference between the branching ratios for the two-body dissociation of $CH_3CH_2OH^{2+}$ and $CD_3CD_2OD^{2+}$. (b) The branching ratios for the two-body dissociation of $CH_3CH_2OH^{2+}$ and $CD_3CD_2OD^{2+}$. Dissociation channels with mass overlaps are combined. Source data are provided as a Source Data file.

As shown in Supplementary Figure 9, the branching ratios for the two-body breakup channels are similar for the CH_3CH_2OH and CD_3CD_2OD isotopologues, as we expected. The small differences are sometimes larger than the experimental uncertainty. To quantify this, we define the significance, S, as the deviation from the mean, i.e. $|R_7 - (R_1 + R_7)/2|$, divided by the sum of the individual experimental uncertainties, which can be expressed as

$$S \equiv \frac{|R_1 - R_7|}{2\sqrt{\sigma_1^2 + \sigma_7^2}}, \tag{S10}$$

where R_1 and R_7 are the branching ratios of the particular breakup channel from the CH_3CH_2OH and CD_3CD_2OD targets, respectively, and σ represents the experimental uncertainty, which is 7× the statistical uncertainty as described in SN 8. The significance of the difference between each two-body breakup channel is shown in Supplementary Figure 9(a), while the branching ratios themselves are shown in Supplementary Figure 9(b). The error bars on S are derived by evaluating the maximum spread of the numerator of Eq. S10 given the experimental uncertainty of R_1 and R_7. The weighted average of S across all the two-body channels is 0.95σ. The isotopic differences for channels with

hydrogen migration, however, tend to be larger than channels associated with simple bond cleavage. To ensure that we have accounted for the uncertainty in the hydrogen migration channels in the determination of the site-specific probabilities, we examine these hydrogen migration channels in more detail.

A. Re-normalization

Since all of the two-body hydrogen migration results are based on the four channels H_3^+ + $C_2H_3O^+$, H_3O^+ + $C_2H_3^+$, H_2O^+ + $C_2H_4^+$, and CH_4^+ + CH_2O^+, we can re-normalize these branching ratios to compare the counts in each channel relative to the total counts in the 3-4 relevant channels, rather than the total counts in all channels. These re-normalized branching ratios R' are given by

$$R_1'\left(\mathrm{H}_3^+\right) = \frac{N\left(\mathrm{H}_3^+ + \mathrm{C}_2\mathrm{H}_3\mathrm{O}^+\right)}{N\left(\mathrm{H}_3^+ + \mathrm{C}_2\mathrm{H}_3\mathrm{O}^+\right) + N\left(\mathrm{H}_3\mathrm{O}^+ + \mathrm{C}_2\mathrm{H}_3^+\right) + N\left(\mathrm{H}_2\mathrm{O}^+ + \mathrm{C}_2\mathrm{H}_4^+\right) + N\left(\mathrm{CH}_4^+ + \mathrm{CH}_2\mathrm{O}^+\right)}. \tag{S11}$$

for the all-H isotopologue and

$$R_7'\left(\mathrm{D}_3^+\right) = \frac{N\left(\mathrm{D}_3^+ + \mathrm{C}_2\mathrm{D}_3\mathrm{O}^+\right)}{N\left(\mathrm{D}_3^+ + \mathrm{C}_2\mathrm{D}_3\mathrm{O}^+\right) + N\left(\mathrm{D}_3\mathrm{O}^+ + \mathrm{C}_2\mathrm{D}_3^+\right) + N\left(\left(\mathrm{D}_2\mathrm{O}^+ + \mathrm{C}_2\mathrm{D}_4^+\right) + \left(\mathrm{CD}_4^+ + \mathrm{CD}_2\mathrm{O}^+\right)\right)}. \tag{S12}$$

for the all-D isotopologue. As in Eq. 1 of the main article, $R_i'(m_1)$ is the re-normalized branching ratio for the m_1 breakup channel of the ith ethanol isotopologue as enumerated in Figure 1 of the main article or Supplementary Table I. Note that, in contrast to Eq. S11, the denominator of Eq. S12 for the completely deuterated isotopologue has only three channels but still represents the same set of fragments as the four channels in the denominator of Eq.S11 due to the mass overlap of D_2O^+ and CD_4^+. The renormalized branching ratios are shown in Supplementary Figure 10, and can be compared for the channels of interest in the same manner as Figure 2 of the main article.

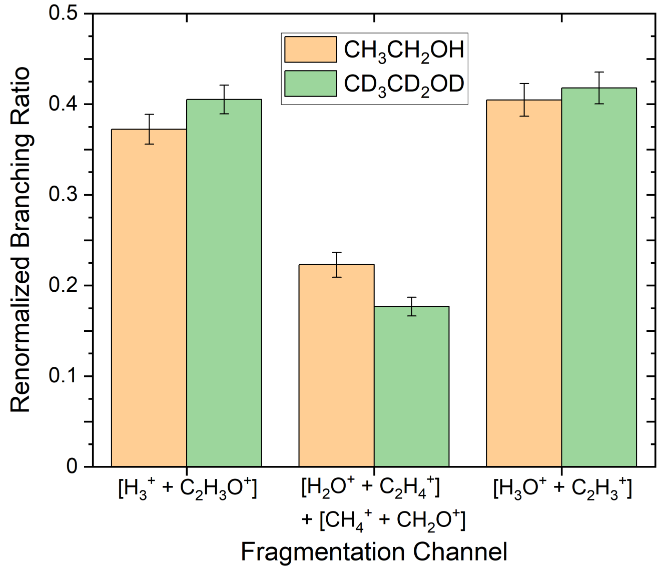

Supplementary Figure 10: Comparison of the re-normalized branching ratios given by Eqs. S11 and S12 for the relevant channels, analagous to Figure 2 of the main article. The error bars reflect the 7× statistical error as described in SN 8. Source data are provided as a Source Data file.

The reduced deviation between the isotopologues in these re-normalized branching ratios as compared to that of the overall branching ratios for the same channels demonstrates that the isotopic effects present are similar in magnitude to the experimental uncertainties in these channels. However, since the overall branching ratios as described by Eq. 1 of the main article are the inputs to the least-squares fit, the comparison of these values in Figure 2 of the main article are more relevant to the overall analysis of the error associated with isotopic effects. Importantly, these re-normalized branching ratios cannot be calculated for all isotopologues since we do not measure all combinations in a 'clean' manner (as described in SN 2) for all isotopologues. Therefore, the re-normalized branching ratios cannot be used in the least-squares fit to determine the site-specific probabilities.

B. Additional Isotopologues

Since not all of the channels are ‘clean’ as described in SN 2, we do not directly measure all of the hydrogen combinations contributing to the formation of each fragment in every isotopologue. This is one reason why the comparison between the pure hydrogen and pure deuterium isotopologues is intuitive, since the $H_3^+ + C_2H_3O^+$ and $D_3^+ + C_2D_3O^+$ channels necessarily represent the full set of H_3^+ combinations. However, any combination of channels which represents exactly the full set of combinations for the fragment of interest can be used to compare isotopic effects. For example, the equations for the $m/q = 5$ and $m/q = 6$ channels in the 6th isotopologue, (listed in Eq. S2 of the SI), are given by

$$\begin{aligned} M5[6] &= \alpha\alpha O + \beta\alpha O + \beta\beta O \\ M6[6] &= \beta\beta\beta + \beta\alpha\alpha + \beta\beta\alpha, \end{aligned} \tag{S13}$$

which together represent all six possible H_3^+ combinations. This means that the sum of these two branching ratios represents exactly the full set of combinations for H_3^+ formation, and can thus be compared to the all-H and all-D branching ratios as in Figure 2 of the main article. Since the $m/q = 4$ channel in the 2nd isotopologue has an identical equation to $M5[6]$, the sum of the $M4[2]$ and $M6[6]$ channels can also be used for comparison. Similarly, the sums of both the $M21[6]$ and $M22[6]$ pair and the $M20[2]$ and $M22[6]$ pair (individual equations listed in Eq. S3 of the SI) represent all six possible H_3O^+ combinations and can be used in the same manner. Only one additional comparison can be made for the H_2O^+ and CH_4^+ channels from the ‘clean’ branching ratios measured, given by the sum of the $M19[6]$ and $M20[6]$ pair (individual equations listed in Eq. S3 of the SI). The resulting branching ratios can be plotted alongside the all-H and all-D isotopologues as shown in Supplementary Figure 11.

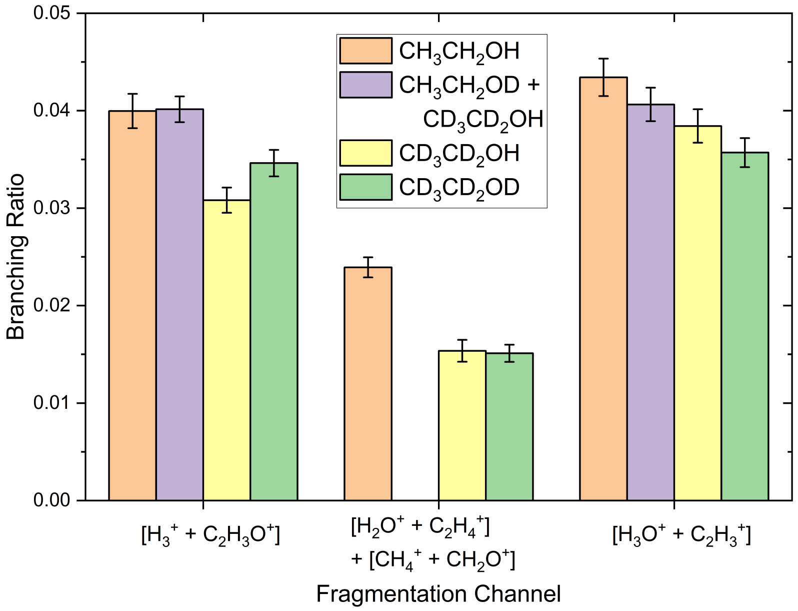

Supplementary Figure 11: Comparison of the all-H and all-D isotopologues alongside additional sums of branching ratios which comprise a full set of combinations for the fragment(s) of interest at various levels of deuteration, analagous to Figure 2 of the main article. The error bars reflect the 7× statistical error as described in SN 8. Source data are provided as a Source Data file.

The decrease in H_3O^+ yield as the level of deuteration increases is consistent with migrating deuterium atoms being less likely to form H_3O^+ than hydrogen atoms. The isotopic results are also consistent with two independent migrations leading to H_3O^+ formation. A similar argument for the other two channels is made more difficult by the potential for more complicated roaming mechanisms and the competing effects of different combinations. Neither of the other two channels follows a consistent dependence with mass, making an attempt to apply a systematic mass correction to the branching ratios difficult.

C. The effect of the least-squares fitting

The overdetermined systems of equations (Eqs. S2, S3, and S5) use information from all seven isotopologues and the equations are not biased toward hydrogen-rich or deuterium-rich isotopes. As a result, the least-squares minimization of the fitting procedure tends to force the solutions toward average values. Since multiple channels representing the same set of hydrogen combinations are used for each product, and each equation has the same weight in the solution, the fit compromises between the two extremes and essentially 'cancels out' isotopic effects. Therefore, the overall effect on the site-specific probabilities is significantly reduced due to the overdetermined system of equations.

The most direct evidence of the effect of the fitting comes from the Monte Carlo technique used to evaluate the uncertainty. Recall that in the Monte Carlo process, random input branching ratios are chosen from a normal distribution centered on the measured branching ratio value. The measurement uncertainty is reflected in the width of the normal distribution of the input. Once the branching ratio inputs needed for the system of equations are randomly selected, the least-squares fit is performed. The process repeats, and the fitting outputs are tabulated and displayed in the violin plots such as Figures 3 and 4 in the main article.

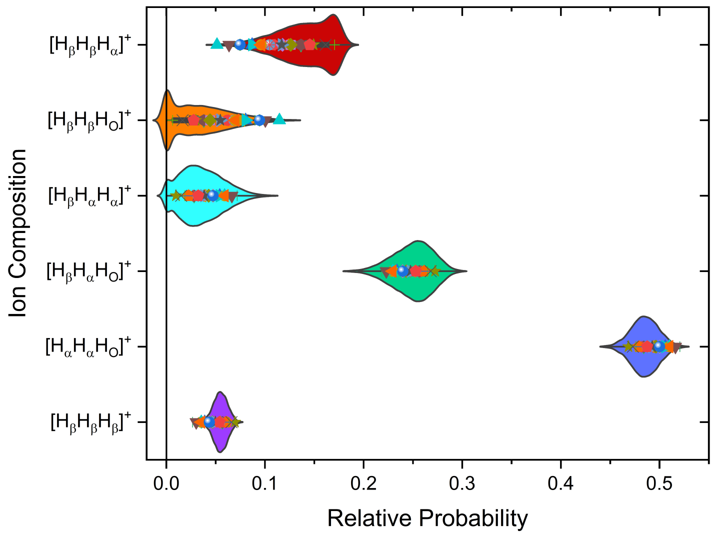

Supplementary Figure 12: The symbols represent the range of least-squares fit outputs after the measured branching ratios are swapped between isotopologues as described in the text. The background violin plot is identical to Figure 3(a) in the main article. Source data are provided as a Source Data file.

By altering various branching ratios artificially, we can estimate the worst-case variation of the site-specific probabilities due to isotopic differences. As discussed in SN 13 B, isotopes #1, #6, and #7 represent all six possible H_3^+ compositions. By swapping the branching ratios for these isotopes in Eq. S2, that is M*[1] ↔ M*[7], M*[1] ↔ M*[6], and M*[6] ↔ M*[7] (where the * represents a placeholder for the appropriate channel), we can then evaluate the range of least-square fits resulting from different combinations. The 27 possible input permutations yield the site-specific probabilities indicated by the symbols in Supplementary Figure 12. The spread of these values is consistent with the range of the Monte Carlo output represented by the shaded kernel density distributions in the background. Moreover, the choice of the violin plot to represent the uncertainty of the fitting results was motivated by the need to show the complexity of propagating the uncertainty through the fits of the systems of equations.

While this analysis has used the H_3^+ system as an example, the conclusions can be generalized to the larger systems of equations S3 and S5. We note that as the systems become more overdetermined, the trend toward the "canceling out" of isotopic effects should become more efficient.

D. Summary

There are measurable differences in the branching ratios obtained from different isotopologues. Overall, these differences are small compared to the overall likelihood of a particular breakup channel; the differences are similar to

measurements in ammonia, methane, and water [16, 17] and smaller than those observed in methanol [18]. Fragmentation channels involving hydrogen migration have a slightly larger isotopic differences than channels that only have bond cleavage. Renormalizing the isotopic differences in comparison to only the hydrogen migration channels illustrates that the isotopic differences are comparable to the relative experimental uncertainty. While the branching ratios follow a consistent trend with mass for H_3O^+ formation, they do not show a simple trend for the formation of H_3^+, H_2O^+, or CH_4^+. If the goal of this project was to determine site-specific isotopic differences for all of the hydrogen migration channels, this would be a concern. Since our goal is to simply determine which hydrogen sites are involved in hydrogen migration processes, these small differences are less of a concern, and the systematic uncertainty, including the isotopic differences, is adequately characterized by the comparison procedure described in SN 8.

SUPPLEMENTARY REFERENCES

[1] N. Ekanayake, T. Severt, M. Nairat, N. P. Weingartz, B. M. Farris, B. Kaderiya, P. Feizollah, B. Jochim, F. Ziaee, K. Borne, K. R. P., K. D. Carnes, D. Rolles, A. Rudenko, B. G. Levine, J. E. Jackson, I. Ben-Itzhak, and M. Dantus, H_2 roaming chemistry and the formation of H_3^+ from organic molecules in strong laser fields, Nature Communications **9**, 10.1038/s41467-018-07577-0 (2018).

[2] S. Zhao, B. Jochim, P. Feizollah, J. Rajput, F. Z. andKanaka Raju P., B. Kaderiya, K. Borne, Y. Malakar, B. Berry, J. Harrington, D. Rolles, A. Rudenko, K. D. Carnes, E. Wells, I. Ben-Itzhak, and T. Severt, Strong-field-induced bond rearrangement in triatomic molecules, Physical Review A **99**, 053412 (2019).

[3] T. Jahnke, T. Weber, T. Osipov, A. Landers, O. Jagutzki, L. Schmidt, C. Cocke, M. Prior, H. Schmidt-Böcking, and R. Dörner, Multicoincidence studies of photo and auger electrons from fixed-in-space molecules using the COLTRIMS technique, Journal of Electron Spectroscopy and Related Phenomena **141**, 229 (2004).

[4] P. Start and R. Parker, Bounded-variable least-squares: an algorithm and applications, Computational Statistics **10**, 129 (1995).

[5] M. A. Branch, T. F. Coleman, and Y. Li, A subspace, interior, and conjugate gradient method for large-scale bound-constrained minimization problems, SIAM Journal on Scientific Computing **21**, 1 (1999).

[6] P. Virtanen, R. Gommers, T. E. Oliphant, M. Haberland, T. Reddy, D. Cournapeau, E. Burovski, P. Peterson, W. Weckesser, J. Bright, S. J. van der Walt, M. Brett, J. Wilson, K. J. Millman, N. Mayorov, A. R. J. Nelson, E. Jones, R. Kern, E. Larson, C. J. Carey, İ. Polat, Y. Feng, E. W. Moore, J. VanderPlas, D. Laxalde, J. Perktold, R. Cimrman, I. Henriksen, E. A. Quintero, C. R. Harris, A. M. Archibald, A. H. Ribeiro, F. Pedregosa, P. van Mulbregt, A. Vijaykumar, A. P. Bardelli, A. Rothberg, A. Hilboll, A. Kloeckner, A. Scopatz, A. Lee, A. Rokem, C. N. Woods, C. Fulton, C. Masson, C. Häggström, C. Fitzgerald, D. A. Nicholson, D. R. Hagen, D. V. Pasechnik, E. Olivetti, E. Martin, E. Wieser, F. Silva, F. Lenders, F. Wilhelm, G. Young, G. A. Price, G.-L. Ingold, G. E. Allen, G. R. Lee, H. Audren, I. Probst, J. P. Dietrich, J. Silterra, J. T. Webber, J. Slavič, J. Nothman, J. Buchner, J. Kulick, J. L. Schönberger, J. V. de Miranda Cardoso, J. Reimer, J. Harrington, J. L. C. Rodríguez, J. Nunez-Iglesias, J. Kuczynski, K. Tritz, M. Thoma, M. Newville, M. Kümmerer, M. Bolingbroke, M. Tartre, M. Pak, N. J. Smith, N. Nowaczyk, N. Shebanov, O. Pavlyk, P. A. Brodtkorb, P. Lee, R. T. McGibbon, R. Feldbauer, S. Lewis, S. Tygier, S. Sievert, S. Vigna, S. Peterson, S. More, T. Pudlik, T. Oshima, T. J. Pingel, T. P. Robitaille, T. Spura, T. R. Jones, T. Cera, T. Leslie, T. Zito, T. Krauss, U. Upadhyay, Y. O. Halchenko, and Y. Vázquez-Baeza, SciPy 1.0: fundamental algorithms for scientific computing in python, Nature Methods **17**, 261 (2020).

[7] G. F. Knoll, *Radiation Detection and Measurement* (John Wiley and Sons Ltd, 2010).

[8] P. R. Bevington, *Data reduction and error analysis for the physical sciences* (McGraw-Hill, 2003) p. 75.

[9] I. Hughes, *Measurements and their uncertainties* (Oxford Univ. Press, 2010) p. 125.

[10] J. Zhang, Modern Monte Carlo methods for efficient uncertainty quantification and propagation: A survey, WIREs Computational Statistics **13**, 10.1002/wics.1539 (2020).

[11] J. Kager and C. Herwig, Monte Carlo-based error propagation for a more reliable regression analysis across specific rates in bioprocesses, Bioengineering **8**, 160 (2021).

[12] J. L. Hintze and R. D. Nelson, Violin plots: A box plot-density trace synergism, American Statistician **52**, 181 (1998).

[13] M. P. Wand and M. Jones, *Kernel smoothing* (Chapman and Hall, 1995).

[14] D. Golomb, R. E. Good, and R. F. Brown, Dimers and clusters in free jets of argon and nitric oxide, The Journal of Chemical Physics **52**, 1545 (1970).

[15] D. Golomb, R. E. Good, A. B. Bailey, M. R. Busby, and R. Dawbarn, Dimers, clusters, and condensation in free jets. II, The Journal of Chemical Physics **57**, 3844 (1972).

[16] B. Jochim, A. Lueking, L. Doshier, S. Carey, E. Wells, E. Parke, M. Leonard, K. D. Carnes, and I. Ben-Itzhak, Rapid formation of H_3^+ from ammonia and methane following 4 MeV proton impact, Journal of Physics B: Atomic, Molecular and Optical Physics **42**, 091002 (2009).

[17] M. Leonard, A. M. Sayler, K. D. Carnes, E. M. Kaufman, E. Wells, R. Cabrera-Trujillo, B. D. Esry, and I. Ben-Itzhak, Bond rearrangement during Coulomb explosion of water molecules, Physical Review A **99**, 012704 (2019).

[18] N. Ekanayake, M. Nairat, B. Kaderiya, P. Feizollah, B. Jochim, T. Severt, B. Berry, Kanaka Raju P., K. D. Carnes, S. Pathak, D. Rolles, A. Rudenko, I. Ben-Itzhak, C. A. Mancuso, B. S. Fales, J. E. Jackson, B. G. Levine, and M. Dantus, Mechanisms and time-resolved dynamics for trihydrogen cation (H_3^+) formation from organic molecules in strong laser fields, Scientific Reports **7**, 10.1038/s41598-017-04666-w (2017).
