# Supplementary material for: Initial-site characterization of hydrogen migration following strong-field double-ionization of ethanol
Source: Nat Commun. 2024 Jan 2;15:74. doi: 10.1038/s41467-023-44311-x (PMC10761976; doi:10.1038/s41467-023-44311-x)
Supplement: Supplementary file 3 — Description of Additional Supplementary Files [file 41467_2023_44311_MOESM3_ESM.pdf]

Description of Additional Supplementary Files

Supplementary Software

Description: The "ethanol_leastsq_mc.py" python source code, which is included as an associated text file, combines the implementation of the least-squares fit and the Monte Carlo uncertainty analysis. The code can be run with any python package. In this case we used Jupyter Notebook. The code contains all the branching ratio input data. Running all three systems with a Monte Carlo size of 10,000 and exporting all the data to delimitated text files takes a Windows-based laptop with a modest CPU around five minutes.
