# Supplementary material for: Initial-site characterization of hydrogen migration following strong-field double-ionization of ethanol
Source: Nat Commun. 2024 Jan 2;15:74. doi: 10.1038/s41467-023-44311-x (PMC10761976; doi:10.1038/s41467-023-44311-x)
Supplement: Supplementary file 5 — Source Data [file 41467_2023_44311_MOESM5_ESM.zip › README Source Data.pdf]

10. SUPPLEMENTARY NOTE 10: EXPLANATION OF THE DATA TABULATED IN THE SOURCE DATA FILES

Tabulated values of the relative probabilities of the initial-site composition of all the ions evaluated in this work are given in the associated spreadsheet file `Source Data - Branching Ratios.xlsx`. These branching ratios form the experimental results that are used to fit the systems of equation described in SN 3-5. To avoid potential character translation issues, the standard Greek letter notation, shown in Supplementary Figure 1 and used throughout the article and the SI, has been altered according to the following scheme:

- $\beta = X$
- $\alpha = Y$
- $O = Z$.

In the first sheet, (`site-specific probabilities`) three different relative probabilities are listed. `Probability relative to three-body` (Column F) is defined using Eq. S6 and is the relative probability used in the article. The associated uncertainty of this relative probability is listed in Column G.

In the case of complete (two-body) fragmentation channels, another reasonable way of defining the relative probabilities is by normalizing the branching ratios to only the sum of complete (two-body) fragmentation channels. For example, the analogous branching ratio to Eq. S6 would now be

$$R_3^{two-body}\left(D_3^+\right) = \frac{N\left(\mathrm{D}_3^+ + \mathrm{C_2H_3O^+}\right)}{\sum_{all} N_C\left(m_1, m_2\right)}, \tag{S7}$$

where $R_i^{two-body}(m_1)$ is the branching ratio for the m_1 breakup channel of the ith ethanol isotopologue as enumerated in SN 1 and $N_C(m_1, m_2)$ is the number of measured ion-pairs from complete channels with m_1 and m_2 being the mass of the first and second ions. This probability is called `Probability relative to two-body` and is listed in Column D, and the associated uncertainty is given in Column E.

Finally, the relative probability of a particular initial-site composition can be determined relative to all possible ion compositions for that fragment. By definition, these probabilities all sum to one for each fragment and are shown in the violin plots. The spreadsheet identifies these as `Probability relative to fragment` in Column B and the associated uncertainty is listed in Column C.

The spreadsheet lists eight sets of probabilities. The description of each data set is listed in Supplementary Table VI.

Supplementary Table VI: Probabilities Listed in Associated Spreadsheet

Label	Description
H3	H_3^+ + $C_2H_3O^+$ complete two-body fragmentation
H3O	H_3O^+ + $C_2H_3^+$ complete two-body fragmentation
H2O	H_2O^+ + $C_2H_4^+$ complete two-body fragmentation
CH4	CH_4^+ + CH_2O^+ complete two-body fragmentation
H3/H	H_3^+ + $C_2H_2O^+$ + H incomplete three-body fragmentation, H initial site not identified
H3/HH	H_3^+ + C_2HO^+ + 2H incomplete three-body fragmentation, 2H initial sites not identified
H3/H(ss)	H_3^+ + $C_2H_2O^+$ + H incomplete three-body fragmentation, H initial site indentified
H3/HH(ss)	H_3^+ + C_2HO^+ + 2H incomplete three-body fragmentation, 2H initial sites identified

The next three sheets contain all of the measured branching ratio data. `2 body (complete) wrt 2 body` has the measured branching ratios with respect to all complete two body fragmentation for all seven isotopologues. Each isotopologue takes up two columns, the first column `BR` is the branching ratio itself, while the second column `BR unc` is the uncertainty. The associated error propagation code multiplies these numbers by seven to get the sum of the statistical and systematic errors as discussed in SN 8. The next sheet `2 body (complete) wrt all counts` is the same data but with respect to the sum of the two-body and three-body counts.

The sheet `3 body wrt all counts` has the branching ratios of interest for the three-body data presented in the main article. Unlike the previous two sheets, these uncertainties represent the sum of the statistical and systematic uncertainty. These three sheets represent the input data needed to create Figures 3 and 4 of the main article using the `ethanol_leastsq_mc.py` python code contained in the supplemental information package.

The file `Source Data - Figures.xlsx` contains the source data for figures 2-5 of the main article. Each dataset is listed in a separate sheet in the Excel spreadsheet. Column headers describe the data, which is almost exclusively probabilities of particular dissociation products.

Likewise, the file `Source Data - Suplementary Figures 3-12.xlsx` contains the source data for Supplementary Figures 3 - 10. Each dataset is listed in a separate sheet in the Excel spreadsheet. The time-of-flight data in sheets `SI-Fig3a`, `SI-Fig3b`, `SI-Fig3c`, `SI-Fig3d` have 2 ns bins. Sheets `SI-Fig7` and `SI-Fig8` have 1 ns bins. For the other sheets, which contain probabilities of particular dissociation products, the data is labelled with headers.

The full CTOF data shown in Supplemental Figure 2 produces an Excel file that is too large for the supplemental information guidelines, so it is saved as a tab-delimited ascii file, `Supplementary Figure 2.txt`. This data has a bin size of 2 ns.
